# Protein Intake and Kidney Outcomes in Nondialysis Chronic Kidney Disease Over 15 Years

**DOI:** 10.1001/jamanetworkopen.2026.9575

**Published:** 2026-04-28

**Authors:** Ilia Beberashvili, Tuvia Baevsky, Dana Shmuel, Israel Yoles, Maya Rosen, Shai Efrati

**Affiliations:** 1 Nephrology Division, Yitzhak Shamir Medical Center, Zerifin, Israel; 2 Clalit Health Services, The Central District, Rishon Le Tzion, Israel

## Abstract

**Importance:**

The optimal dietary protein intake (DPI) for patients with chronic kidney disease (CKD) remains uncertain. Long-term, clinical practice data using objective DPI measures are limited, and concerns about nutritional risk persist.

**Objective:**

To evaluate the association of objectively measured DPI with long-term kidney and clinical outcomes in adults with CKD stages 3 and 4.

**Design, Setting, and Participants:**

This retrospective cohort study included adults with CKD stages 3 and 4 receiving care in Clalit Health Services, Israel, with index entry from January 1, 2007, through December 31, 2022, and follow-up of up to 15 years. DPI was assessed using 24-hour urinary nitrogen excretion and normalized to adjusted body weight (normalized DPI [nDPI]). Participants were stratified by an nDPI threshold of 1.0 g/kg/d (selected using time-dependent receiver operating characteristic analysis). Data were analyzed from August to October 2025.

**Exposure:**

nDPI calculated from 24-hour urinary nitrogen excretion, analyzed dichotomously using the 1.0 g/kg/d threshold.

**Main Outcomes and Measures:**

The primary outcome was a composite of 50% or more decline in estimated glomerular filtration rate (eGFR), initiation of long-term dialysis, or all-cause mortality. Secondary outcomes included individual components and trajectories of eGFR and albuminuria. Propensity score matching was used to balance baseline characteristics between lower (<1.0 g/kg/d) and higher (≥1.0 g/kg/d) nDPI groups. Kidney function was evaluated using mixed-effects models and joint models linking eGFR trajectories with time-to-event outcomes.

**Results:**

Of 1441 included patients (mean [SD] age, 67.20 [11.26] years; 507 [35.2%] women), 530 were matched (265 per group). During follow-up, the lower-nDPI group had a lower risk of the composite outcome (hazard ratio [HR], 0.77; 95% CI, 0.62-0.97; log-rank *P* = .03), mainly related to fewer dialysis initiations (HR, 0.65; 95% CI, 0.42-0.99). In adjusted Cox models, low nDPI remained associated with lower composite risk (HR, 0.75; 95% CI, 0.60-0.93). Longitudinal models showed no significant between-group differences in eGFR or albuminuria slopes; eGFR decline was numerically slower in the low-nDPI group (slope difference, 0.152 mL/min/1.73 m^2^/y). No differences in nutritional markers were observed.

**Conclusions and Relevance:**

In this retrospective cohort study of adults with CKD stages 3 and 4, lower nDPI (<1.0 g/kg/d) was associated with lower dialysis risk without nutritional harm. These findings support moderate protein restriction with routine DPI monitoring in CKD care.

## Key Points

**Question:**

Is lower dietary protein intake (DPI), measured by 24-hour urine collections, associated with favorable outcomes in adults with stages 3 and 4 chronic kidney disease (CKD)?

**Findings:**

In this cohort study of 1441 adults, normalized DPI (nDPI; grams per kilogram body weight per day) was estimated from 24-hour urinary nitrogen excretion. In matched analyses, nDPI less than 1.0 was associated with lower risk of the composite outcome (≥50% estimated glomerular filtration rate decline, dialysis initiation, or death) and dialysis initiation, without meaningful differences in nutritional markers.

**Meaning:**

These findings suggest that moderate protein intake (nDPI <1.0 g/kg per day) with objective monitoring may be associated with favorable kidney outcomes in routine CKD care.

## Teaser

This cohort study evaluates the association of objectively measured dietary protein intake with long-term kidney and clinical outcomes in adults with stages 3 and 4 chronic kidney disease (CKD).

## Introduction

Chronic kidney disease (CKD) management has advanced greatly with therapies such as sodium-glucose cotransporter–2 (SGLT-2) inhibitors and glucagon-like peptide–1 (GLP-1) receptor agonists, which have demonstrated renoprotective and survival benefits.^1,2,3,4^ However, despite these pharmacological breakthroughs, patients continue to experience estimated glomerular filtration rate (eGFR) decline, proteinuria, and high cardiovascular risk. Nutritional strategies, particularly regulation of dietary protein intake (DPI), remain underutilized in addressing this residual risk.

Although landmark trials such as DAPA-CKD,^1^ EMPA-Kidney,^2^ CREDENCE,^3^ FLOW,^4^ and FIGARO-DKD^5^ have reshaped nephrology, none reported participants’ dietary intake. This omission reflects a broader oversight in both clinical trials and routine care, where nutritional factors are often neglected. Consequently, a persistent gap remains between dietary guidelines^6,7^ and clinical practice.

Several factors contribute to this gap. First, actual DPI in populations with CKD frequently exceeds the recommended 0.8 g per kilogram of body weight (BW) per day, as shown in US,^8^ European,^9^ and Asian cohorts.^10^ Second, randomized trials have produced mixed results,^11,12,13,14,15^ often with short follow-up^13,14,15^ or small samples,^15^ limiting conclusions about long-term outcomes. Third, many observational studies lack serial or objective DPI assessments, relying on nonsystematic methods^16,17^ or onetime baseline data.^18^ Finally, barriers to implementation, including variable assessment tools, cultural norms, and adherence, further complicate translation into practice.^19^

To address these limitations, we conducted a 15-year retrospective cohort study of patients with stages 3 and 4 CKD in Israel. Using 24-hour urinary nitrogen excretion to objectively monitor DPI, we evaluated the long-term association between protein intake and CKD progression, dialysis initiation, and mortality under clinical practice conditions.

## Methods

### Data Sources

This retrospective cohort study was part of the DISP-CKD (Dietary Interventions and Slowing the Progression of CKD) project evaluating dietitian-supported CKD nutrition care. It was approved by the institutional review board and the Helsinki Committee of Clalit Health Services (CHS), with informed consent waived because of the study design. CHS is Israel’s largest managed care organization, serving over 2.1 million individuals across central, northern, and southern districts. Since 1998, it has maintained a comprehensive electronic medical database containing demographic, clinical, and laboratory data. The study period was January 1, 2002, to December 31, 2022. For the present normalized DPI (nDPI) analysis, participants entered the cohort between January 1, 2007, and December 31, 2022, allowing up to 15 years of follow-up. Reporting followed the Strengthening the Reporting of Observational Studies in Epidemiology (STROBE) reporting guidelines for cohort studies.

### Study Population and Data Collection

We included adults (aged ≥18 years) with CKD stages 3 and 4, defined by 2 eGFR values between 15 and 60 mL/min/1.73 m^2^ at least 90 days apart, per Kidney Disease Improving Global Outcomes guidelines.^7^ Exclusion criteria were eGFR greater than 60 or less than 15 mL/min/1.73 m^2^, dialysis or kidney transplant, active malignant disease, or missing data for DPI estimation (eg, 24-hour urine creatinine, urea, or protein). Patients with incomplete urine collections—determined by comparing measured vs estimated 24-hour creatinine excretion according to age, sex, weight, and height—were excluded, as were those with fluctuating DPI who could not be consistently categorized.

**Figure 1.**
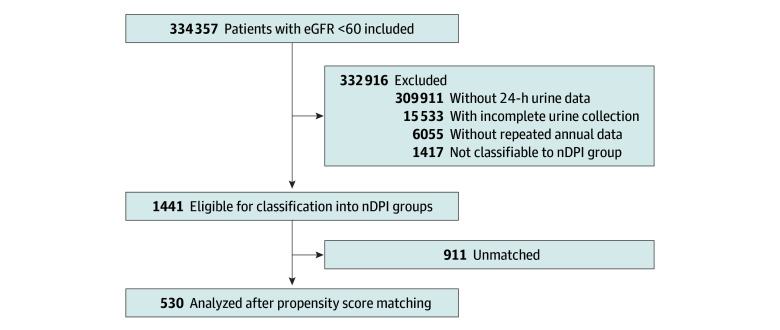
Flowchart of Patient Selection The unit of measure for estimated glomerular filtration rate (eGFR) is milliliters per minutes per 1.73 m^2^. nDPI indicates normalized dietary protein intake.

Baseline demographic and clinical data were recorded at study entry. Annual follow-up data were collected for up to 15 years or until death or dialysis. For patients with multiple annual measurements, values were averaged. All included participants received at least 1 dietitian consultation with standard counseling on protein restriction (0.8 g/kg/d); follow-up counseling was variable and not protocol mandated, and analyses focused on achieved nDPI. Eligibility required at least 3 usable 24-hour urine collections over follow-up (to enable longitudinal assessment of nDPI), with sufficient data to verify collection adequacy; this selection yielded a cohort with more male patients and more advanced CKD than excluded patients (eTable 1 in Supplement 1).^3^ The patient selection process is outlined in Figure 1. Urine collections were clinically driven (routine care) rather than protocol mandated; when performed, they were typically recommended approximately 2 to 4 times per year depending on CKD severity, but actual frequency varied. Repeated adequate collections are often not obtained in routine practice (eg, inconsistent ordering, incomplete collections, and occasional laboratory issues); therefore, serial usable collections were available for only a minority of CHS patients with CKD.

### Variables

Baseline data included age, sex, blood pressure, body mass index (BMI; calculated as weight in kilograms divided by height in meters squared), Geriatric Nutritional Risk Index (GNRI) score, and laboratory values (serum creatinine, albumin, hemoglobin, hemoglobin A_1c_ [HbA_1c_]; and urinary urea nitrogen, creatinine, protein, sodium, and urine albumin-to-creatinine ratio [UACR]). Medication use (angiotensin-converting enzyme inhibitor [ACEi] or angiotensin receptor blocker [ARB], statins, SGLT-2 inhibitors, and GLP-1 receptor agonists) was extracted from pharmacy data; adherence was defined as 75% or more of the prescribed annual dose.

eGFR was extracted from the institutional database, where values were routinely calculated using the Modification of Diet in Renal Disease (MDRD) equation.^20^ During the earlier study years, creatinine assays were not consistently isotope dilution mass spectrometry–standardized, precluding Chronic Kidney Disease Epidemiology Collaboration application.

Nutritional status was assessed via the GNRI,^21^ as recommended by the Kidney Disease Outcomes Quality Initiative,^6^ where GNRI = [14.89 × albumin (g/dL)] + [41.7 × (weight / ideal weight)], with ideal weight calculated using sex-specific Lorentz equations as described by Bouillanne et al.^21^ GNRI has been validated in both dialysis^22^ and nondialysis^23^ CKD populations. During follow-up, annual updates were collected for creatinine, albumin, BMI, GNRI, UACR, urinary parameters, and medication use.

### DPI Calculation

We assessed the adequacy of 24-hour urine collections by comparing measured creatinine excretion with values estimated using the following equation: estimated creatinine (mg/d) = 699 − 421.9 (if female) + 16.84 × weight (kg) − 25.82 (if White) − 2.67 × age (years).^24^ Patients below this threshold were excluded. DPI was calculated using 24-hour urea urinary nitrogen per the Maroni et al^25^ formula: DPI = 6.25 × [urea urinary nitrogen (g) + 0.031 × weight (kg)] + proteinuria (g), where 6.25 reflects that protein is approximately 16% nitrogen.^26^ Including proteinuria accounts for nitrogen loss in nephrotic-range cases (5.6% before matching). This approach has been previously applied in clinical nephrology settings.^18,27^ To ensure robustness, all models adjusted for baseline proteinuria and sensitivity analyses excluded the proteinuria term.

To account for excess weight, DPI was normalized to adjusted BW calculated according to the Kidney Disease Outcomes Quality Initiative^6^: adjusted BW = ideal BW + [(actual BW − ideal BW) × 0.25], with ideal weight calculated by the Lorentz method, as described by Bouillanne et al.^21^ DPI was then expressed as nDPI in grams per kilogram BW per day.

### Comorbidity Index and Clinical Outcomes

Comorbidity burden was assessed using the Charlson Comorbidity Index.^28^ The primary composite outcome was time to first occurrence of (1) 50% or greater eGFR decline confirmed 28 or more days later, (2) dialysis initiation (no patients underwent preemptive kidney transplantation), or (3) all-cause death. Secondary outcomes included each component of the primary outcome, analyzed individually and hierarchically.

### Laboratory Methods

All laboratory tests were performed at CHS central laboratories during outpatient visits. Serum creatinine was measured by the kinetic Jaffe method, measuring albumin by bromocresol green. Urinary analytes (creatinine, urea, protein, sodium, and albumin) were analyzed from 24-hour collections. Complete blood count including hemoglobin and HbA_1c_ were measured by automated systems. All data reflect routine clinical care.

### Statistical Analysis

Data were analyzed from August to October 2025. Continuous variables are presented as mean (SD) or median (IQR), as appropriate, on the basis of variable distribution (approximately normal vs skewed), and categorical variables are presented as counts and percentages. The 95% CIs are presented for model-based estimates (hazard ratios [HRs], slopes, and spline or joint-model outputs). Between-group comparisons used *t* tests or Mann-Whitney *U* tests for continuous variables, and χ^2^ tests for categorical variables.

To identify the optimal baseline nDPI cutoff, time-dependent receiver operating characteristic analyses were performed. A 1.0 g/kg/d threshold was selected on the basis of performance in survival and longitudinal models and alignment with dietary guidelines.^6,7^

To minimize baseline imbalances between nDPI groups (<1.0 vs ≥1.0 g/kg/d), we used propensity score matching (PSM) via logistic regression. Covariates included age, sex, baseline eGFR, systolic blood pressure, Charlson Comorbidity Index score, BMI, GNRI, albumin, hemoglobin, UACR, urinary sodium, and medication use (ACEi or ARBs, statins, GLP-1 agonists, and SGLT-2 inhibitors). Nearest-neighbor matching with a 0.1 SD caliper was applied. Matching quality was assessed using standardized mean differences (SMDs), with SMD less than 0.1 indicating balance.

Time-to-event outcomes (composite, dialysis, 50% eGFR decline, and death) were analyzed using Kaplan-Meier curves and Cox proportional hazards models. Patients were censored at 180 months or at competing events.

The proportional hazards assumption was assessed using Schoenfeld residuals and inspection of residual plots. To address potential information loss from dichotomization, baseline nDPI was also modeled as a continuous exposure (per 0.2 g/kg/d) in adjusted Cox models, and restricted cubic splines were used to assess potential nonlinearity. As a sensitivity analysis to examine potential time-varying associations, we fit extended Cox models with an nDPI × log(time + 1) interaction and robust SEs clustered by participant.

Joint models were built to analyze longitudinal eGFR changes with clinical events, combining linear mixed-effects and Cox models.^29^ Covariates were selected on the basis of PSM cohort SMDs. Joint models accounted for informative dropout due to clinical events.

Missing longitudinal measurements were handled using multiple imputation by chained equations in long format. Missingness was modest (≤10% per variable; nDPI, <7%; proteinuria and 24-hour sodium, approximately 6%; UACR, approximately 3%; and eGFR, approximately 2%). The imputation model included follow-up year, baseline covariates, treatment group, and available longitudinal biomarkers; predictive mean matching was used for skewed variables (eg, nDPI, UACR, proteinuria, and 24-hour sodium). Imputation was performed only within observed follow-up and not beyond each participant’s censoring or event time. Imputed datasets were used for longitudinal trajectory analyses and joint models; baseline Cox or spline analyses used observed baseline values. Analyses were conducted across imputed datasets and combined using Rubin rules; complete-case analyses yielded similar conclusions. All analyses were conducted in R statistical software version 4.4.2 (R Project for Statistical Computing) and SPSS statistical software version 29.0 (IBM). A 2-sided *P* < .05 was considered statistically significant.

## Results

### Baseline Characteristics

We included 1441 patients with CKD stages 3 and 4 (mean [SD] age, 67.20 [11.26] years; 507 [35.2%] women). Median (IQR) follow-up was 67 (34-112) months. Incidence rates were 42.4 per 1000 person-years for 50% eGFR decline, 27.8 per 1000 person-years for dialysis initiation, and 52.9 per 1000 person-years for all-cause death. The median (IQR) baseline nDPI was 1.18 (1.02-1.36) g/kg/d (eFigure 1 in Supplement 1), with most patients consuming above the recommended dietary allowance of 0.8 g/kg/d.

### Baseline nDPI as a Continuous Exposure

**Figure 2.**
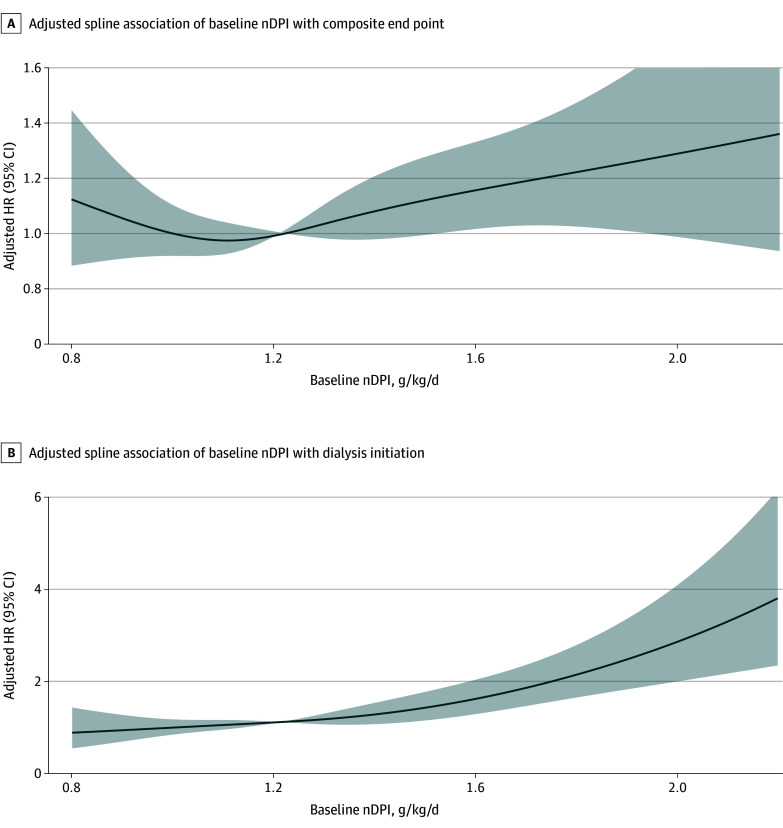
Line Graphs of Adjusted Spline Association of Baseline Normalized Dietary Protein Intake (nDPI) With the Composite End Point (Full Cohort) Graphs show adjusted hazard ratios (HRs; solid lines) and 95% CIs (shaded areas) for the composite end point (dialysis initiation, 50% estimated glomerular filtration rate decline, or all-cause death) (A) and for dialysis initiation (B) across baseline nDPI modeled using restricted cubic splines in the eligible cohort (n = 1441). Models were adjusted for age, sex, diabetes status, Charlson Comorbidity Index score, systolic blood pressure, chronic kidney disease stage, angiotensin-converting enzyme inhibitor or angiotensin receptor blocker use, statin use, sodium-glucose cotransporter–2 inhibitor use, and glucagon-like peptide–1 receptor agonist use. The reference nDPI value was 1.0 g/kg/d. Estimates at nDPI less than 0.8 g/kg/d should be interpreted cautiously because of sparse data.

Using the full eligible cohort of 1441 patients, we fit multivariable Cox models with baseline nDPI (per 0.2 g/kg/d) as the exposure and time to first composite event as the outcome; dialysis initiation, all-cause death, and 50% eGFR decline were also modeled separately, adjusted for prespecified baseline covariates (eTable 2 in Supplement 1; covariates are described in the Methods). Higher nDPI was associated with higher risk of the composite outcome (adjusted HR, 1.05; 95% CI, 1.00-1.10; *P* = .04) and dialysis initiation (adjusted HR, 1.13; 95% CI, 1.06-1.21; *P* < .001) (eTable 2 in Supplement 1). Restricted cubic spline models showed no strong evidence of nonlinearity, with risk lowest at approximately 1.0 g/kg/d; estimates at nDPI less than 0.8 g/kg/d should be interpreted cautiously owing to limited observations (Figure 2). In sensitivity analyses allowing the association between nDPI and outcomes to vary over time, an extended Cox model with an nDPI × log(time + 1) interaction suggested higher risk with longer follow-up per 0.2 g/kg/d higher nDPI (eTable 3 in Supplement 1).

### nDPI Cutoff Selection and PSM

Time-dependent receiver operating characteristic analyses identified optimal nDPI cutoffs for outcome estimation (eFigure 2 in Supplement 1). On the basis of these and prior modeling considerations (see Statistical Analysis), a threshold of 1.0 g/kg/d was chosen. Patients were categorized as low (<1.0 g/kg/d; 321 patients) or high (≥1.0 g/kg/d; 1120 patients) nDPI. Over 15 years, protein intake declined significantly in the high nDPI group (−0.019 g/kg/d/y; 95% CI, −0.0304 to −0.0083 g/kg/d; *P* < .001), yet remained above 1.0 g/kg/d. In the low group, intake remained stable (−0.008 g/kg/d/y; 95% CI, −0.0186 to 0.0027 g/kg/d/y; *P* = .14) (eFigure 3 in Supplement 1).

Table 1 shows baseline characteristics before and after PSM. Prematching differences included diabetes, BMI, HbA_1c_, UACR, proteinuria, ACEi or ARB use, and statin use. After matching (530 patients; 265 patients per group), groups were well balanced (SMDs <0.1), including age, sex, comorbidities, eGFR, GNRI, albumin, albuminuria, proteinuria, and medications. Balance improvements are shown in eFigure 4 in Supplement 1.

**Table 1.** Baseline Demographic, Clinical, and Laboratory Characteristics of the Study Population and PSM Cohort, Stratified by nDPI Adjusted by Weight^a^

Variable	Original cohort (N = 1441)			PSM cohort (n = 530)^b^		
	Patients, No. (%)		SMD^c^	Patients, No. (%)		SMD^c^
	nDPI <1.0 g/kg/d (n = 321)	nDPI ≥1.0 g/kg/d (n = 1120)		nDPI <1.0 g/kg/d (n = 265)	nDPI ≥1.0 g/kg/d (n = 265)	
Age, mean (SD), y	67.0 (12.0)	67.2 (11.5)	0.02	67.6 (11.4)	67.2 (12.0)	0.03
Sex						
Male	193 (60.1)	741 (66.2)	0.13	169 (63.8)	166 (62.6)	0.02
Female	128 (39.9)	379 (33.8)		96 (36.2)	99 (37.4)	
Geographic region						
Center	210 (65.4)	645 (57.6)	0.19	161 (60.8)	182 (68.7)	0.22
North	18 (5.6)	34 (3.0)		7 (2.6)	13 (4.9)	
South	93 (29.0)	441 (39.4)		97 (36.6)	70 (26.4)	
Ethnic group						
Jewish	302 (94.1)	034 (92.3)	0.07	248 (93.6)	252 (95.1)	0.06
Arab	19 (5.9)	86 (7.7)		17 (6.4)	13 (4.9)	
Charlson Comorbidity Index score, median (IQR)	5 (3-7)	5 (3-7)	0.1	5 (3-7)	5 (3-7)	0.00
Diabetes (yes)	120 (37.4)	594 (53.0)	0.32	107 (40.4)	109 (41.1)	0.01
Systolic blood pressure, mean (SD), mm Hg	130.1 (10.6)	131.0 (10.7)	0.09	129.7 (9.7)	130.2 (10.7)	0.05
Body mass index, mean (SD)^d^	27.0 (4.4)	29.1 (4.7)	0.47	27.3 (4.2)	27.2 (4.3)	0.02
Geriatric Nutritional Risk Index score, mean (SD)	103.5 (5.0)	103.5 (5.3)	0.01	103.8 (5.4)	103.5 (4.9)	0.04
Albumin, mean (SD), g/dL	4.2 (0.3)	4.2 (0.3)	0.05	4.2 (0.3)	4.2 (0.3)	0.05
Creatinine, median (IQR), mg/dL	1.29 (1.00-1.51)	1.41 (1.14-1.80)	0.05	1.35 (1.10-1.60)	1.30 (1.06-1.54)	0.01
Phosphorus, mean (SD), mg/dL	3.70 (0.65)	3.71 (0.72)	0.02	3.68 (0.72)	3.69 (0.67)	0.01
Estimated glomerular filtration rate, mean (SD), mL/min/1.73 m^2^	42.7 (11.3)	43.0 (11.5)	0.03	43.4 (11.3)	43.1 (11.4)	0.03
Chronic kidney disease stage						
G3a	157 (48.9)	527 (47.1)	0.01	132 (49.8)	137 (51.7)	0.08
G3b	111 (34.6)	421 (37.1)		97 (36.6)	87 (32.8)	
G4	53 (16.5)	172 (15.4)		36 (13.6)	41 (15.5)	
Hemoglobin, mean (SD), g/dL	12.3 (1.6)	12.5 (1.8)	0.1	12.3 (1.8)	12.3 (1.6)	0.03
Hemoglobin A_1C_, mean (SD), %	6.6 (0.7)	6 .9 (1.1)	0.33	6.5 (0.7)	6.6 (0.7)	0.06
Urinary sodium/24 h, median (IQR), mEq	160 (118-206)	163 (121-214)	0.02	163 (120-209)	159 (119-207)	0.01
Urine albumin-to-creatinine ratio, median (IQR), mg/g	48 (0-314)	96 (0-413)	0.29	37 (0-290)	30 (0-233)	0.02
Proteinuria ≥1 g/d	29 (9.0)	312 (27.9)	0.48	32 (12.1)	27 (10.2)	0.06
Angiotensin-converting enzyme inhibitor or angiotensin receptor blocker (yes)	145 (45.2)	683 (61.0)	0.32	124 (46.8)	131 (49.4)	0.05
Statin (yes)	120 (37.4)	589 (52.6)	0.31	102 (38.5)	106 (40.0)	0.03
Sodium-glucose cotransporter–2 inhibitor (yes)	30 (9.3)	97 (8.7)	0.02	24 (9.1)	27 (10.2)	0.04
Glucagon-like peptide–1 receptor agonist (yes)	6 (1.9)	18 (1.6)	0.02	3 (1.1)	5 (1.9)	0.06

Abbreviations: nDPI, normalized dietary protein intake; PSM, propensity score matched; SMD, standardized mean difference.

SI conversion factors: To convert albumin to grams per liter, multiply by 10; creatinine to micromoles per liter, multiply by 88.4; hemoglobin to grams per liter, multiply by 10; hemoglobin A_1C _to proportion of total hemoglobin, multiply by 0.01; phosphorus to millimoles per liter, multiply by 0.323.

^a^ nDPI was calculated using 24-hour urinary urea nitrogen and normalized to adjusted body weight.

^b^ The PSM cohort was created using 1:1 nearest-neighbor matching without replacement.

^c^ An SMD greater than or equal to 0.1 was considered indicative of meaningful imbalance.

^d^ Body mass index is calculated as weight in kilograms divided by height in meters squared.

### Survival Analyses in PSM Cohort

**Figure 3.**
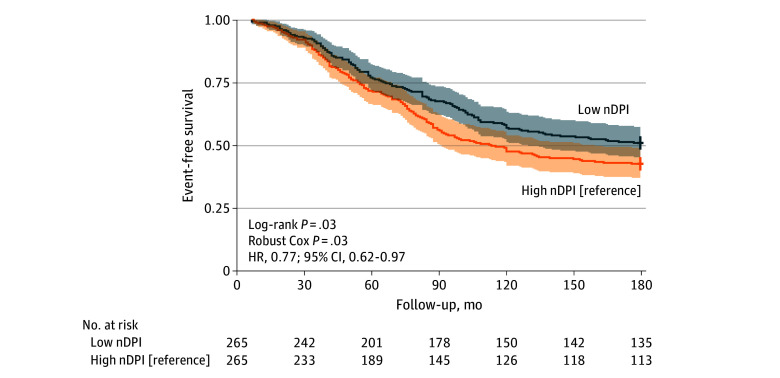
Kaplan-Meier Survival Curves for the Composite Outcome in the Propensity Score–Matched Cohort, Stratified by Normalized Dietary Protein Intake (nDPI) Patients in the low nDPI group (<1.0 g/kg/d) had a significantly lower incidence of the composite outcome compared with those in the high nDPI group (≥1.0 g/kg/d). Numbers at risk are shown. Estimates at late follow-up should be interpreted cautiously owing to reduced numbers at risk. Note, in our study, low nDPI refers to moderately restricted protein intake (<1.0 g/kg/d), rather than a classic low-protein diet (0.6-0.8 g/kg/d). We, therefore, use the term *low nDPI* throughout to denote this moderate level of restriction.

In the PSM cohort of 530 patients, we compared low vs high nDPI for time-to-first event outcomes using Kaplan-Meier log-rank and Cox models (robust SEs), with follow-up censored at 180 months. Kaplan-Meier curves showed a lower risk of the composite outcome in the low nDPI group compared with the high nDPI reference group (HR, 0.77; 95% CI, 0.62-0.97; log-rank *P* = .03; robust Cox *P* = .03) (Figure 3). This association was primarily related to dialysis initiation, for which the low nDPI group showed a lower risk (HR, 0.65; 95% CI, 0.42-0.99; log-rank *P* = .06; robust Cox *P* = .04) (eFigure 5A in Supplement 1). By contrast, associations for 50% eGFR decline and all-cause death were directionally consistent but did not reach statistical significance (eFigures 5B and 5C in Supplement 1). Numbers at risk are shown below each plot; interpretation of later follow-up should remain cautious as the risk set declines over time.

Multivariable Cox models adjusting for key covariates showed that low nDPI remained associated with lower risk of the composite outcome (model 1, HR, 0.75; 95% CI, 0.60-0.93; *P* = .01; model 2, HR, 0.78; 95% CI, 0.62-0.97; *P* = .03) (Table 2). Results for individual outcomes were directionally consistent; the association reached statistical significance for dialysis initiation, whereas associations with 50% eGFR decline and all-cause death were not statistically significant (Table 2).

**Table 2.** Multivariable Cox Proportional Hazards Models in the Propensity Score–Matched Cohort for Estimating the Composite Outcome of 50% eGFR Decline, KRT Initiation, or All-Cause Death in Patients With Chronic Kidney Disease

Outcome	No. of events (n = 530)	Model 1^a^		Model 2^b^	
		HR (95% CI)	*P* value	HR (95% CI)	*P* value
Composite outcome^c^	282	0.75 (0.60-0.93)	.01	0.78 (0.62-0.97)	.03
KRT initiation	77	0.57 (0.36-0.92)	.02	0.61 (0.38-0.98)	.04
All-cause death	161	0.87 (0.63-1.18)	.37	0.89 (0.65-1.22)	.48
50% eGFR decline	152	0.77 (0.57-1.04)	.08	0.80 (0.59-1.09)	.15

Abbreviations: eGFR, estimated glomerular filtration rate; HR, hazard ratio; KRT, kidney replacement therapy.

^a^ Model 1 includes age, sex, diabetes, body mass index, eGFR, serum albumin, and urine albumin-to-creatinine ratio.

^b^ Model 2 includes the same covariates as model 1, but replaces urine albumin-to-creatinine ratio with 24-hour proteinuria.

^c^ The composite outcome includes 50% decline in eGFR, initiation of KRT, or all-cause death.

In the PSM cohort, proportional hazards assumptions were met for nDPI group. The significant global test was primarily related to Charlson Comorbidity Index score (eFigures 6A and 6B in Supplement 1).

Subgroup analyses (eFigure 7 in Supplement 1) demonstrated consistent associations across age, sex, ethnic groups, region, diabetes, CKD stage, albuminuria, proteinuria, systolic blood pressure, BMI, and medication use. Subgroup HRs were estimated from Cox models including the interaction tested by adding nDPI group by subgroup terms. All interaction *P* values were not significant. The overall HR for low vs high nDPI was 0.71 (95% CI, 0.55-0.93), supporting a robust association.

### Longitudinal Analysis

In the matched cohort, we modeled annual eGFR and change in UACR as repeated outcomes using linear mixed-effects models with fixed effects for time, nDPI group, and time by group and random intercepts (eFigures 8A and 8B in Supplement 1). Joint models then linked each longitudinal biomarker trajectory to time-to-event outcomes (composite and components; eTables 4-7 in Supplement 1) by combining the mixed-effects submodel with a Cox survival submodel. In longitudinal mixed-effects models, eGFR declined over time in both nDPI groups; the low nDPI group showed a numerically less steep eGFR slope (slope difference [low minus high], 0.152 mL/min/1.73 m^2^/y), although the group-by-time interaction was not significant (eFigure 8A in Supplement 1), consistent with limited precision after PSM. The difference in UACR increased over follow-up in both groups with no significant difference in slopes (eFigure 8B in Supplement 1). In joint models (eTables 4-7 in Supplement 1), time-updated biomarker levels were consistently associated with outcomes in expected directions: higher contemporaneous kidney injury markers (eg, albuminuria or proteinuria and phosphorus) tracked higher risk, whereas better nutritional markers (eg, albumin or GNRI) tracked lower risk across end points. Overall, these analyses indicate that event risk closely follows longitudinal disease burden, whereas between-group trajectory differences in the PSM cohort were not statistically significant.

## Discussion

In this large, 15-year longitudinal cohort of patients with nondialysis CKD stages 3 and 4, we observed that sustained moderate protein intake (nDPI <1.0 g/kg/d), assessed by 24-hour urinary nitrogen excretion, was associated with a reduced risk of adverse clinical outcomes, particularly dialysis initiation. This benefit was consistent across clinical subgroups. These findings suggest that modest protein restriction, even above traditionally recommended thresholds, may confer renal protection without compromising nutritional status in CKD management in clinical practice. The observed incidence rates of 50% eGFR decline, dialysis initiation, and all-cause death align with those reported in other longitudinal CKD studies, indicating that our cohort reflects typical progression risk in stage 3 and 4 CKD.^7^

Whether dietary protein restriction slows CKD progression remains controversial. Randomized trials have produced mixed results, with some supporting low-protein diet benefits^11,12,13,30,31,32,33,34,35^ and others showing no effect.^14,15,36^ These inconsistencies likely reflect small samples,^15,31,32,33^ heterogeneous dietary targets (0.8 g/kg/d^12,36^ to very-low-protein diets <0.6 g/kg/d with or without ketoanalogues^14,15,16,32,33^), less rigorous designs than landmark studies like MDRD,^31,32,33,34,35^ and heterogeneity in study populations (eg, elderly,^32^ nondiabetic,^12,32^ and unselected^13,14,15,16,33,34,35,36^ cohorts). Many trials also lacked patient-centered approaches and did not report adherence. Observational studies have similarly yielded conflicting results, with some supporting^10,17,25^ and others not supporting^16,18^ low-protein diet benefits. Despite larger samples and longer follow-up, most lacked systematic and serial DPI monitoring,^10,16,17,18,37^ making adherence a persistent uncertainty. Our study addresses these gaps through regular 24-hour urinary nitrogen assessments over 15 years, enabling evaluation of clinical practice intake patterns and adherence.

Choosing an appropriate nDPI threshold is another key consideration. In this observational study, we used a clinically interpretable cutoff aligned with prior CKD nutrition literature and guidelines.^6,7^ To address concerns about dichotomization, we also examined nDPI as a continuous exposure and with spline functions, with similar overall inferences. Although interventional trials typically recommend 0.6 to 0.8 g/kg/d,^6,11,38,39^ observational cohorts have suggested a wider and more flexible range.^19,37,40^ In our cohort, the optimal threshold emerged around 1.0 g/kg/d, consistent with findings from several observational studies.^41^ Lower thresholds (0.8 or 0.6 g/kg/d) could not be evaluated because of the relatively high protein intake in our population. Interestingly, the large interventional study by Locatelli et al^36^ showed no difference in CKD progression between 0.6 and 1.0 g/kg/d, supporting the plausibility of a higher threshold. The mean nDPI in our study was similar to that reported in the study by Cirillo et al,^9^ further supporting external validity. Collectively, these findings reinforce 1.0 g/kg/d as a pragmatic target for Israeli patients with CKD.

We observed no evidence of nutritional harm associated with lower protein intake, supporting the safety of modest dietary protein restriction. These results align with interventional and observational studies showing that diets in the 0.6 to 0.8 g/kg/d range can be safely implemented when energy intake is adequate and monitoring is consistent.^11,13,32,42^ Concerns regarding nutritional risk largely stem from studies involving very-low-protein diets (eg, 0.3 g/kg/d), particularly in elderly or highly comorbid populations.^43,44^ Given the complexity of CKD diets, our findings suggest that a moderate reduction in protein intake may offer a balanced, safe, and feasible approach.

Moderate nDPI (<1.0 g/kg/d) was associated with reduced risk of dialysis initiation and the composite outcome of dialysis, 50% eGFR decline, or death during long-term follow-up. Although the risk reduction was smaller than that observed with recent pharmacologic interventions, such as SGLT-2 inhibitors or mineralocorticoid receptor antagonists,^1,2,5^ it remains clinically meaningful and highlights the potential role of dietary intervention in addressing residual renal risk.

Another observation is the underuse of 24-hour urinary nitrogen monitoring in routine CKD care. Despite being the most accurate noninvasive tool for assessing DPI, its use appears limited, as demonstrated by the proportion of patients excluded due to missing 24-hour urine data. This likely reflects broader deficiencies in individualized dietary management in Israeli CKD care. Similar trends have been reported globally, with underuse of structured dietary interventions and objective monitoring methods.^19,45,46^

### Strengths and Limitations

Strengths of this study include the large, well-characterized CKD cohort, extended follow-up, and objective DPI assessment using 24-hour urinary nitrogen excretion. The clinical practice nature of the data enhances clinical relevance.

Several limitations merit consideration. First, the retrospective design may introduce selection bias and unmeasured confounding. Selection bias is a key limitation because 24-hour urine collections are obtained in a minority of patients with CKD in routine care. Included patients differed from excluded patients (eTable 1 in Supplement 1), and, therefore, the findings may be most generalizable to patients with CKD managed in settings where timed urine collections are routinely performed. In addition, the effective sample size decreased over follow-up due to censoring and events; therefore, estimates at later time points (particularly beyond approximately 10 years) are less precise and should be interpreted cautiously. Second, despite using objective DPI measurements, missing 24-hour urine values accounted for less than 10% of all annual observations, and these were handled using multiple imputation to minimize potential bias. Third, we lacked detailed information on dietary prescriptions (eg, food-based recommendations), counseling intensity, and adherence, which limits translation of our findings into specific guidance regarding which foods and what amounts. Fourth, eGFR was calculated using the MDRD equation, consistent with laboratory reporting during the study period, and MDRD performs comparably to the Chronic Kidney Disease Epidemiology Collaboration in patients with eGFR less than 60 mL/min/1.73 m^2^.^47^ Fifth, we lacked data on protein type (animal vs plant) and did not include dietary recall assessments, which were not systematically collected; instead, we relied on 24-hour urinary urea nitrogen to minimize recall bias. Future studies should combine urine-based intake estimates with standardized dietary assessment (eg, repeated 24-hour recalls or food records or validated food frequency questionnaires) capturing protein source or quality and key conutrients (eg, sodium, potassium, and phosphate) to enable actionable, food-level recommendations. In addition, given the 2007 to 2022 study period, uptake of contemporary therapies (particularly SGLT-2 inhibitors and GLP-1 receptor agonists) was limited for much of follow-up, which may limit generalizability to current CKD practice.

## Conclusions

In this retrospective cohort study of adults with CKD stages 3 and 4, lower nDPI (<1.0 g/kg/d)—reflecting moderate protein restriction—was associated with a lower risk of the composite outcome, primarily related to fewer dialysis initiations. Given the pragmatic, clinical practice approach to monitoring intake, these findings may be especially relevant to clinical settings where long-term adherence and feasibility are key considerations.

## Notes

Supplement 1. eTable 1. Baseline characteristics of included (n=1,441) vs excluded (n=23,005) patients with at least 1 urine collection during the follow up period eTable 2. Association of baseline nDPI (continuous) with study outcomes in Cox proportional hazards models eTable 3. Time-varying association of nDPI with the composite outcome and dialysis initiation (extended Cox models) eTable 4. Longitudinal Trajectories of Nutritional and Kidney Function Biomarkers by nDPI Group and Their Associations with the Composite Outcome in Joint Modeling eTable 5. Longitudinal Trajectories of Nutritional and Kidney Function Biomarkers by nDPI Group and Their Associations with Dialysis Initiation in Joint Modeling eTable 6. Longitudinal Trajectories of Nutritional and Kidney Function Biomarkers by nDPI Group and Their Associations with All-cause Death in Joint Modeling eTable 7. Longitudinal Trajectories of Nutritional and Kidney Function Biomarkers by nDPI Group and Their Associations with 50% eGFR Decline in Joint Modeling eFigure 1. Histogram of baseline normalized dietary protein intake (nDPI) in the study population (n = 1,441) eFigure 2. Time-dependent area under the curve (AUC) for baseline normalized dietary protein intake (nDPI) in predicting clinical outcomes over 15 years of follow-up eFigure 3. Longitudinal trajectories of normalized dietary protein intake (nDPI) over 15 years of follow-up, stratified by baseline nDPI (<1.0 vs. ≥1.0 g/kg/day) eFigure 4. Standardized mean differences (SMDs) for baseline covariates before and after propensity score matching eFigure 5. Kaplan–Meier survival curves for dialysis initiation and all-cause mortality by nDPI group eFigure 6. Scaled Schoenfeld residuals for the nDPI exposure and Charlson comorbidity score (composite outcome) in the propensity score–matched cohort eFigure 7. Subgroup analysis of the association between normalized dietary protein intake (nDPI) and the composite outcome (50% decline in eGFR, dialysis initiation, or all-cause death) in the propensity score-matched cohort eFigure 8. Mean eGFR trajectories and geometric mean changes in urine albumin-to-creatinine ratio (ΔUACR) over 15 years of follow-up in patients with low (<1.0 g/kg/day) and high (≥1.0 g/kg/day) normalized dietary protein intake (nDPI), with 95% confidence intervals

Supplement 2. Data Sharing Statement
