# Error in Figure 3

**DOI:** 10.1001/jamanetworkopen.2026.20366

**Published:** 2026-06-02

In the Original Investigation titled “Protein Intake and Kidney Outcomes in Nondialysis Chronic Kidney Disease Over 15 Years,”^1^ published April 28, 2026, there was an error in Figure 3. The labels for the lines for high normalized dietary protein intake and low normalized dietary protein intake in the graph and the numbers at risk were switched. This article has been corrected.^1^
