# Supplementary material for: Protein Intake and Kidney Outcomes in Nondialysis Chronic Kidney Disease Over 15 Years
Source: JAMA Netw Open. 2026 Apr 28;9(4):e269575. doi: 10.1001/jamanetworkopen.2026.9575 (PMC13126224; doi:10.1001/jamanetworkopen.2026.9575)
Supplement: Supplement 1. — eTable 1. Baseline characteristics of included (n=1,441) vs excluded (n=23,005) patients with at least 1 urine collection during the follow up period eTable 2. Association of baseline nDPI (continuous) with study outcomes in Cox proportional hazards models eTable 3. Time-varying association of nDPI with the composite outcome and dialysis initiation (extended Cox models) eTable 4. Longitudinal Trajectories of Nutritional and Kidney Function Biomarkers by nDPI Group and Their Associations with the Composite Outcome in Joint Modeling eTable 5. Longitudinal Trajectories of Nutritional and Kidney Function Biomarkers by nDPI Group and Their Associations with Dialysis Initiation in Joint Modeling eTable 6. Longitudinal Trajectories of Nutritional and Kidney Function Biomarkers by nDPI Group and Their Associations with All-cause Death in Joint Modeling eTable 7. Longitudinal Trajectories of Nutritional and Kidney Function Biomarkers by nDPI Group and Their Associations with 50% eGFR Decline in Joint Modeling eFigure 1. Histogram of baseline normalized dietary protein intake (nDPI) in the study population (n = 1,441) eFigure 2. Time-dependent area under the curve (AUC) for baseline normalized dietary protein intake (nDPI) in predicting clinical outcomes over 15 years of follow-up eFigure 3. Longitudinal trajectories of normalized dietary protein intake (nDPI) over 15 years of follow-up, stratified by baseline nDPI (<1.0 vs. ≥1.0 g/kg/day) eFigure 4. Standardized mean differences (SMDs) for baseline covariates before and after propensity score matching eFigure 5. Kaplan–Meier survival curves for dialysis initiation and all-cause mortality by nDPI group eFigure 6. Scaled Schoenfeld residuals for the nDPI exposure and Charlson comorbidity score (composite outcome) in the propensity score–matched cohort eFigure 7. Subgroup analysis of the association between normalized dietary protein intake (nDPI) and the composite outcome (50% decline in eGFR, dialysis initiation, or all-cause [file jamanetwopen-e269575-s001.pdf]

Supplemental Online Content

Beberashvili I, Baevsky T, Shmuel D, Yoles I, Rosen M, Efrati S. Protein intake and kidney outcomes in nondialysis chronic kidney disease over 15 years. *JAMA Netw Open*. 2026;9(4):e269575. doi:10.1001/jamanetworkopen.2026.9575

eTable 1. Baseline characteristics of included (n=1,441) vs excluded (n=23,005) patients with at least 1 urine collection during the follw up period

eTable 2. Association of baseline nDPI (continuous) with study outcomes in Cox proportional hazards models

eTable 3. Time-varying association of nDPI with the composite outcome and dialysis initiation (extended Cox models)

eTable 4. Longitudinal Trajectories of Nutritional and Kidney Function Biomarkers by nDPI Group and Their Associations with the Composite Outcome in Joint Modeling

eTable 5. Longitudinal Trajectories of Nutritional and Kidney Function Biomarkers by nDPI Group and Their Associations with Dialysis Initiation in Joint Modeling

eTable 6. Longitudinal Trajectories of Nutritional and Kidney Function Biomarkers by nDPI Group and Their Associations with All-cause Death in Joint Modeling

eTable 7. Longitudinal Trajectories of Nutritional and Kidney Function Biomarkers by nDPI Group and Their Associations with 50% eGFR Decline in Joint Modeling

eFigure 1. Histogram of baseline normalized dietary protein intake (nDPI) in the study population (n = 1,441)

eFigure 2. Time-dependent area under the curve (AUC) for baseline normalized dietary protein intake (nDPI) in predicting clinical outcomes over 15 years of follow-up

eFigure 3. Longitudinal trajectories of normalized dietary protein intake (nDPI) over 15 years of follow-up, stratified by baseline nDPI (<1.0 vs. ≥1.0 g/kg/day)

eFigure 4. Standardized mean differences (SMDs) for baseline covariates before and after propensity score matching

eFigure 5. Kaplan–Meier survival curves for dialysis initiation and all-cause mortality by nDPI group

eFigure 6. Scaled Schoenfeld residuals for the nDPI exposure and Charlson comorbidity score (composite outcome) in the propensity score–matched cohort

eFigure 7. Subgroup analysis of the association between normalized dietary protein intake (nDPI) and the composite outcome (50% decline in eGFR, dialysis initiation, or all-cause death) in the propensity score-matched cohort

eFigure 8. Mean eGFR trajectories and geometric mean changes in urine albumin-to-creatinine ratio (ΔUACR) over 15 years of follow-up in patients with low (<1.0 g/kg/day) and high (≥1.0 g/kg/day) normalized dietary protein intake (nDPI), with 95% confidence intervals

This supplemental material has been provided by the authors to give readers additional information about their work.

© 2026 Beberashvili I et al. *JAMA Network Open*.

eTable 1. Baseline characteristics of included (n=1,441) vs excluded (n=23,005) patients with at least 1 urine collection during the follw up period

Variable	Included	Excluded	SMD
Age, mean (SD), y	67.20 (11.26)	69.10 (12.29)	0.16
Sex, n (%)			0.35
Male	934 (64.8)	11 011 (47.9)	
Female	507 (35.2)	11 994 (52.1)	
Region, n (%)			0.72
• Center	855 (59.3)	11 286 (49.1)	
• North	52 (3.6)	6 406 (27.8)	
• South	534 (37.1)	5 313 (23.1)	
Ethnicity, n (%)			0.11
• Jew			
• Arab	105 (7.3)	2 369 (10.3)	
Charlson CS, median [IQR]	5.00 [3.00, 7.00]	6.00 [5.00, 8.00]	0.32
Diabetes, n (%)	714 (49.5)	9 603 (41.7)	0.16
SBP, mean (SD), mm Hg	130.83 (10.66)	134.03 (20.31)	0.20
BMI, mean (SD), kg/m²	28.63 (4.69)	30.57 (8.65)	0.28
GNRI, mean (SD)	103.48 (5.24)	101.32 (7.40)	0.34
Albumin, mean (SD), g/dL	4.16 (0.34)	3.98 (0.52)	0.41
Creatinine, median [IQR]	1.40 [1.10, 1.70]	1.21 [1.01, 1.45]	0.04
Phosphorus, mean (SD)	3.71 (0.70)	3.66 (0.81)	0.07
eGFR, mean (SD), mL/min/1.73m²	42.90 (11.45)	49.27 (11.30)	0.56
CKD stage, n (%)			0.53

© 2026 Beberashvili I et al. *JAMA Network Open*.

• G3a	666 (46.8)	14 152 (71.0)	
• G3b	532 (37.4)	4 651 (23.3)	
• G4	225 (15.8)	1 139 (5.7)	
Hemoglobin, mean (SD), g/dL	12.42 (1.75)	12.68 (1.62)	0.15
HbA1c, mean (SD), %	6.81 (1.04)	7.15 (1.62)	0.25
24-hour urinary sodium, median [IQR]	162 [120, 211]	Not available	-
UACR, median [IQR]	85.22 [0.00, 460.94]	Not available	-
24-h urinary protein g/24h, median [IQR]	0.30 [0.14, 0.93]	Not available	-
ACEi/ARB use, n (%)	828 (57.5)	10 378 (45.1)	0.25
Statin use, n (%)	709 (49.2)	8 426 (36.6)	0.26
SGLT2 inhibitor use, n (%)	127 (8.8)	334 (1.5)	0.34
GLP-1RA use, n (%)	24 (1.7)	124 (0.5)	0.11e

Footnote: SMD indicates standardized mean difference; values >0.10 suggest meaningful imbalance.

Brief interpretation: eTable 1 compares included and excluded patients with at least 1 urine collection during follow-up. Differences are summarized using standardized mean differences (SMDs) rather than P values given the large sample size. Included patients differed from excluded patients across several characteristics, including sex distribution, CKD severity (eGFR and stage), comorbidity burden, and nutritional indices, consistent with selection related to availability of complete urine-based dietary assessment.

© 2026 Beberashvili I et al. *JAMA Network Open*.

eTable 2. Association of baseline nDPI (continuous) with study outcomes in Cox proportional hazards models

Outcome	Events/N	unadjusted HR (95% CI)	P	Adjusted HR (95% CI)	P
Composite outcome	861/1441	1.09 (1.050-1.13)	<.001	1.05 (1.00-1.10)	.04
Dialysis initiation	294/1441	1.18 (1.12-1.26)	<.001	1.13 (1.06-1.21)	<.001
All-cause death	563/1441	1.05 (1.00-1.11)	.03	1.04 (0.98-1.10)	.19
50% eGFR decline	432/1441	1.07 (1.01-1.13)	.03	1.03 (0.96-1.10)	.41

Exposure: baseline nDPI, modeled continuously (hazard ratio [HR] per 0.2 g/kg/day higher nDPI).

Adjusted model covariates: age, sex, diabetes status, Charlson comorbidity score, systolic blood pressure, CKD stage, ACEi/ARB use, statin use, SGLT-2 inhibitor use, GLP-1 receptor agonist use.

Notes: Composite endpoint defined as time to first of dialysis initiation, 50% eGFR decline, or all-cause death.

Abbreviations: CKD, chronic kidney disease; eGFR, estimated glomerular filtration rate; HR, hazard ratio; nDPI, normalized dietary protein intake; SBP, systolic blood pressure.

© 2026 Beberashvili I et al. *JAMA Network Open*.

eTable 3. Time-varying association of nDPI with the composite outcome and dialysis initiation (extended Cox models)

Follow-up time (months)	Composite outcome HR (95% CI)	Dialysis initiation HR (95% CI)
36	1.02 (1.01–1.03)	1.05 (1.04–1.06)
60	1.04 (1.02–1.07)	1.10 (1.07–1.13)
120	1.08 (1.03–1.12)	1.17 (1.12–1.23)
180	1.10 (1.04–1.16)	1.22 (1.16–1.29)

Footnote: Model: Time-specific HRs were derived from the nDPI × log(time+1) interaction in the extended Cox model (time in months). Robust standard errors were clustered by participant to account for start–stop (time-updated) intervals. Effect estimate: Hazard ratio (HR) per +0.2 g/kg/day higher nDPI, evaluated at prespecified follow-up times.

© 2026 Beberashvili I et al. *JAMA Network Open*.

eTable 4. Longitudinal Trajectories of Nutritional and Kidney Function Biomarkers by nDPI Group and Their Associations with the Composite Outcome in Joint Modeling

Marker	Trajectory	Longitudinal submodel		Survival submodel	
	Component	Estimate (95% CI)	P value	HR (95% CI)	P value
eGFR (ml/min/1.73m^2)					
	Intercept	51.56 (48.35 to 54.76)	<.001	0.93 (0.91–0.94)	<.001
	Slpoe per year	−1.23 (−1.45 to −1.02)	<.001		
	∆Slope	0.11 (−0.19 to 0.41)	.55		
BMI (kg/m^2)					
	Intercept	24.46 (22.08 to 26.84)	<.001	0.98 (0.96–1.01)	.28
	Slope per year	−0.10 (−0.13 to −0.06)	<.001		
	∆Slope	0.02 (−0.04 to 0.07)	.55		
GNRI					
	Intercept	107.54 (105.29 to 109.78)	<.001	0.87 (0.82–0.93)	<.001
	Slope per year	−0.49 (−0.56 to −0.42)	<.001		
	∆Slope	0.02 (−0.08 to 0.12)	.69		
Systolic blood pressure (mm Hg)					
	Intercept	119.1 (113.2 to 125.0)	<.001	1.01 (1.00 to 1.04)	.04
	Slope per year	0.07 (−0.11 to 0.25)	.44		
	∆Slope	-0.08 (−0.34 to 0.17)	.54		
Albumin (g/dL)					
	Intercept	4.50 (4.36 to 4.64)	<.001	0.10 (0.04–0.25)	<.001
	Slpoe per year	−0.032 (−0.037 to −0.028)	<.001		
	∆Slope	0.002 (−0.008 to 0.005)	.76		

© 2026 Beberashvili I et al. *JAMA Network Open*.

Phosporus (mg/dl)				
Intercept	3.69 (3.39 to 3.98)	<.001	2.24 (1.76–2.88)	<.001
Slope per year	0.02 (0.01 to 0.04)	<.001		
∆Slope	-0.02 (−0.04 to -0.002)	.04		
UACR (mg/g)				
Intercept	4.47 (3.55 to 5.20)	<.001	1.34 (1.19–1.51)	<.001
Slpoe per year	0.11 (0.08 to 0.14)	<.001		
∆Slope	−0.025 (−0.019 to 0.069)	.67		
24h Proteinuria (g/day)				
Intercept	0.66 (0.50 to 0.83)	<.001	1.214 (1.11–1.32)	<.001
Slpoe per year	1.0002 (0.00005 to 0.0004)	.001		
∆Slope	−0.00009 (−0.0003 to 0.0001)	.47		

Estimates from the longitudinal submodel include baseline levels (intercepts), yearly change (slopes), and slope differences (∆Slope) between low and high normalized dietary protein intake (nDPI) groups. High normalized dietary protein intake (nDPI ≥ 1.0 g/kg/day) was used as the reference group. ΔSlope indicates the difference in yearly change (slope) between the Low nDPI group (<1.0 g/kg/day) and the High nDPI group. Positive values indicate slower decline or greater increase in the Low nDPI group. Hazard ratios (HR) reflect the association of each time-varying biomarker with the composite outcome (50% decline in eGFR, dialysis initiation, or all-cause death). Models adjusted for age, gender, diabetes status, Charlson comorbidity score, and CKD stage.

© 2026 Beberashvili I et al. *JAMA Network Open*.

eTable 5. Longitudinal Trajectories of Nutritional and Kidney Function Biomarkers by nDPI Group and Their Associations with Dialysis Initiation in Joint Modeling

Marker	Trajectory	Longitudinal submodel		Survival submodel	
	Cpmponent	Estimate (95% CI)	P value	HR (95% CI)	P value
eGFR (ml/min/1.73m^2)					
	Intercept	51.89 (48.73 to 55.07)	<.001	0.81 (0.79–0.84)	<.001
	Slope per year	−1.38 (−1.55 to −1.31)	<.001		
	∆Slope	0.17 (-0.14 to 0.48)	.28		
BMI (kg/m^2)					
	Intercept	24.12 (21.89 to 26.55)	<.001	1.00 (0.95–1.05)	.97
	Slope per year	−0.08 (−0.21 to −0.05)	<.001		
	∆Slope	-0.02 (−0.07 to 0.04)	.52		
GNRI					
	Intercept	106.99 (104.94 to 109.13)	<.001	0.87 (0.82–0.93)	<.001
	Slope per year	−0.47 (−0.54 to −0.40)	<.001		
	∆Slope	−0.003 (−0.10 to 0.10)	.95		
Systolic blood pressure (mm Hg)					
	Intercept	120.1 (114.6 to 126.0)	<.001	1.08 (1.02 to 1.15)	.003
	Slope per year	0.01 (−0.14 to 0.16)	.88		
	∆Slope	0.05 (−0.21 to 0.28)	.65		
Albumin (g/dL)					
	Intercept	4.48 (4.35 to 4.63)	<.001	0.10 (0.04–0.25)	<.001
	Slope per year	−0.030 (−0.035 to −0.026)	<.001		
	∆Slope	−0.001 (−0.0084 to 0.005)	.76		

© 2026 Beberashvili I et al. *JAMA Network Open*.

Phosphorus (mg/dl)				
Intercept	3.69 (3.41 to 3.96)	<.001	4.29 (2.42 to 7.31)	<.001
Slope per year	0.003 (−0.009 to 0.017)	.62		
∆Slope	0.02 (0.003 to 0.04)	.03		
UACR (mg/g)				
Intercept	354.1 (290.9 to 415.3)	<.001	1.0000 (0.9997–1.0031)	.97
Slope per year	47.9 (19.8 to 76.3)	<.001		
∆Slope	−16.4 (−50.7 to 17.8)	.36		
24h Proteinuria (g/day)				
Intercept	1.93 (1.30 to 2.61)	<.001	1.29 (1.19 to 1.39)	<.001
Slpoe per year	0.08 (0.02 to 0.13)	.004		
∆Slope	−0.05 (−0.12 to 0.03)	.20		

Estimates from the longitudinal submodel include baseline levels (intercepts), yearly change (slopes), and slope differences (∆Slope) between low and high normalized dietary protein intake (nDPI) groups. High normalized dietary protein intake (nDPI ≥ 1.0 g/kg/day) was used as the reference group. ΔSlope indicates the difference in yearly change (slope) between the Low nDPI group (<1.0 g/kg/day) and the High nDPI group. Positive values indicate slower decline or greater increase in the Low nDPI group. Hazard ratios (HR) reflect the association of each time-varying biomarker with the composite outcome (50% decline in eGFR, dialysis initiation, or all-cause death). Models adjusted for age, gender, diabetes status, Charlson comorbidity score, and CKD stage.

© 2026 Beberashvili I et al. *JAMA Network Open*.

eTable 6. Longitudinal Trajectories of Nutritional and Kidney Function Biomarkers by nDPI Group and Their Associations with All-cause Death in Joint Modeling

Marker	Trajectory Cpmponent	Longitudinal submodel Estimate (95% CI)	P value	Survival submodel HR (95% CI)	P value
eGFR (ml/min/1.73m^2)					
	Intercept	51.76 (48.92 to 54.68)	<.001	0.91 (0.86–0.96)	<.001
	Slope per year	−0.24 (−1.46 to −1.04)	<.001		
	∆Slope	-0.19 (−0.50 to 0.12)	.22		
BMI (kg/m^2)					
	Intercept	24.12 (21.90 to 26.55)	<.001	1.00 (0.95–1.05)	.97
	Slope per year	-0.08 (−0.12 to −0.05)	<.001		
	∆Slope	-0.02 (−0.07 to 0.047)	.52		
GNRI					
	Intercept	106.99 (104.94 to 109.13)	<.001	0.87 (0.82–0.93)	<.001
	Slope per year	-0.47 (−0.54 to −0.40)	<.001		
	∆Slope	-0.003 (−0.10 to 0.10)	.95		
Systolic blood pressure (mm Hg)					
	Intercept	120.1 (114.6 to 126.0)	<.001	1.08 (1.02–1.15)	.003
	Slope per year	0.01 (−0.14 to 0.16)	.80		
	∆Slope	0.05 (−0.21 to 0.28)	.65		
Albumin (g/dL)					
	Intercept	4.88 (4.35 to 4.63)	<.001	0.10 (0.04–0.25)	<.001
	Slope per year	−0.030 (−0.035 to −0.026)	<.001		
	∆Slope	0.0001 (−0.006 to 0.005)	.76		

© 2026 Beberashvili I et al. *JAMA Network Open*.

Phosphorus (mg/dl)				
Intercept	3.69 (3.41 to 3.96)	<.001	1.01 (0.99 to 1.03)	.40
Slope per year	0.003 (−0.009 to 0.017)	.62		
∆Slope	0.02 (0.003 to 0.039)	.03		
UACR (mg/g)				
Intercept	235.8 (201.4 to 270.2)	<.001	1.000 (1.000–1.0001)	.008
Slope per year	33.9 (27.1 to 40.7)	<.001		
∆Slope	−6.63 (−18.49 to 5.46)	.27		
24h Proteinuria (g/day)				
Intercept	1.73 (1.22 to 2.28)	<.001	1.40 (1.26 to 1.55)	<.001
Slope per year	0.08 (0.03 to 0.13)	.001		
∆Slope	−0.03 (−0.10 to 0.04)	.35		

Estimates from the longitudinal submodel include baseline levels (intercepts), yearly change (slopes), and slope differences (∆Slope) between low and high normalized dietary protein intake (nDPI) groups. High normalized dietary protein intake (nDPI ≥ 1.0 g/kg/day) was used as the reference group. ΔSlope indicates the difference in yearly change (slope) between the Low nDPI group (<1.0 g/kg/day) and the High nDPI group. Positive values indicate slower decline or greater increase in the Low nDPI group. Hazard ratios (HR) reflect the association of each time-varying biomarker with the composite outcome (50% decline in eGFR, dialysis initiation, or all-cause death). Models adjusted for age, gender, diabetes status, Charlson comorbidity score, and CKD stage.

© 2026 Beberashvili I et al. *JAMA Network Open*.

eTable 7. Longitudinal Trajectories of Nutritional and Kidney Function Biomarkers by nDPI Group and Their Associations with 50% eGFR Decline in Joint Modeling

Marker	Trajectory Cpmponent	Longitudinal submodel Estimate (95% CI)	P value	Survival submodel HR (95% CI)	P value
eGFR (ml/min/1.73m²)					
	Intercept	52.08 (49.24 to 54.99)	<.001	0.99 (0.99–0.99)	<.001
	Slope per year	-1.20 (−1.41 to −1.00)	<.001		
	∆Slope	-0.16 (−0.46 to 0.15)	.30		
BMI (kg/m²)					
	Intercept	24.24 (21.79 to 26.56)	<.001	0.99 (0.95–1.03)	.66
	Slope per year	−0.08 (−0.12 to −0.04)	<.001		
	∆Slope	- 0.02 (−0.07 to 0.03)	.56		
GNRI					
	Intercept	106.96 (104.71 to 109.19)	<.001	0.95 (0.91–0.99)	.04
	Slope per year	−0.47 (−0.54 to −0.40)	<.001		
	∆Slope	0.002 (−0.10 to 0.11)	.56		
Systolic blood pressure (mm Hg)					
	Intercept	120.0 (114.5 to 125.8)	<.001	1.01 (0.98–1.04)	.54
	Slope per year	0.003 (−0.15 to 0.17)	.97		
	∆Slope	0.013 (−0.24 to 0.27)	.92		
Albumin (g/dL)					
	Intercept	4.47 (4.34 to 4.62)	<.001	0.44 (0.20–0.91)	.02
	Slope per year	−0.030 (−0.035 to −0.026)	<.001		
	∆Slope	−0.0006 (−0.0-7 to 0.006)	.86		

© 2026 Beberashvili I et al. *JAMA Network Open*.

Phosphorus (mg/dl)				
Intercept	3.69 (3.39 to 3.97)	<.001	1.63 (1.01 to 2.51)	.047
Slope per year	0.003 (−0.01 to 0.02)	.72		
∆Slope	0.02 (0.003 to 0.04)	.03		
UACR (mg/g)				
Intercept	235.8 (201.4 to 270.2)	<.001	1.0008 (1.0004–1.0012)	.009
Slope per year	33.9 (27.1 to 40.7)	<.001		
∆Slope	Not reported			
24h Proteinuria (g/day)				
Intercept	1.72 (1.21 to 2.27)	<.001	1.26 (1.11 to 1.41)	.002
Slpoe per year	0.08 (0.03 to 0.13)	<.001		
∆Slope	−0.03 (−0.10 to 0.04)	.40		

Estimates from the longitudinal submodel include baseline levels (intercepts), yearly change (slopes), and slope differences (∆Slope) between low and high normalized dietary protein intake (nDPI) groups. High normalized dietary protein intake (nDPI ≥ 1.0 g/kg/day) was used as the reference group. ΔSlope indicates the difference in yearly change (slope) between the Low nDPI group (<1.0 g/kg/day) and the High nDPI group. Positive values indicate slower decline or greater increase in the Low nDPI group. Hazard ratios (HR) reflect the association of each time-varying biomarker with the composite outcome (50% decline in eGFR, dialysis initiation, or all-cause death). Models adjusted for age, gender, diabetes status, Charlson comorbidity score, and CKD stage.

© 2026 Beberashvili I et al. *JAMA Network Open*.

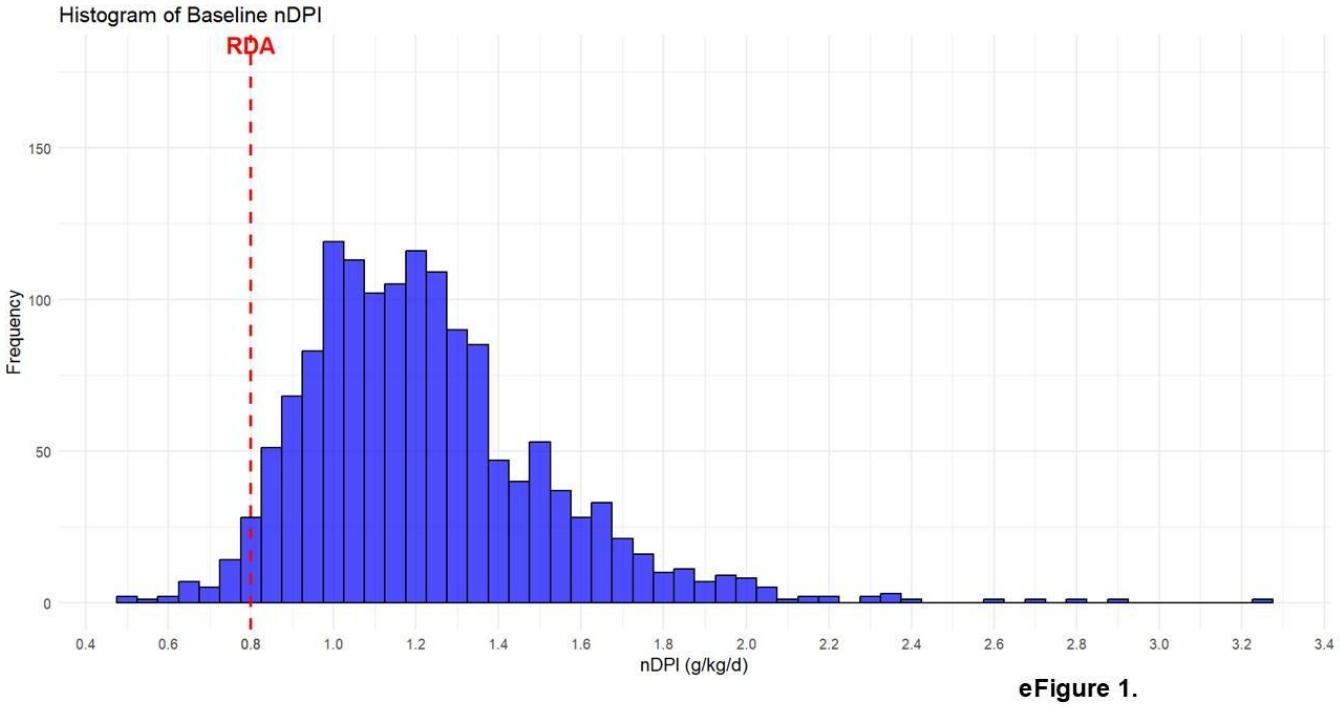

eFigure 1. Histogram of baseline normalized dietary protein intake (nDPI) in the study population (n = 1,441). The dashed red line indicates the recommended dietary allowance (RDA) for protein intake in CKD, set at 0.8 g/kg/day. Given mild right-skewness, nDPI is summarized as median [IQR] in the Results.

© 2026 Beberashvili I et al. *JAMA Network Open*.

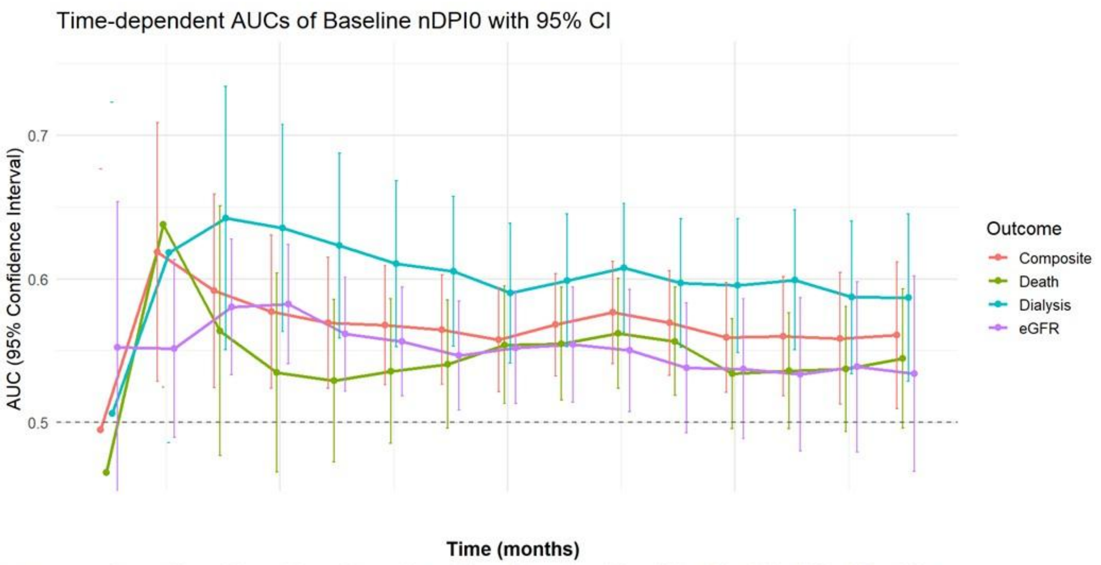

Outcome	0	12	24	36	48	60	72	84	96	108	120	132	144	156	168	180
Composite outcome	1441	1326	1198	1063	916	799	693	599	493	396	318	265	210	167	120	85
50% eGFR decline	1441	1328	1200	1064	916	799	693	599	493	396	318	265	210	167	120	85
Dialysis initiation	1441	1361	1286	1212	1116	1020	935	843	733	621	521	436	349	275	204	154
All-cause death	1441	1369	1306	1249	1174	1095	1025	946	841	736	630	551	457	363	270	202

eFigure 2.

eFigure 2. Time-dependent area under the curve (AUC) for baseline normalized dietary protein intake (nDPI) in predicting clinical outcomes over 15 years of follow-up. Each coloured line represents a different outcome: composite event, dialysis initiation, all-cause death, and ≥50% decline in estimated glomerular filtration rate (eGFR). Points denote AUC values at each year, with vertical error bars representing 95% confidence intervals. The dashed grey line indicates an AUC of 0.5 (no discrimination). Overall, nDPI0 showed most stable discrimination for dialysis initiation, with consistent performance across other outcomes over time.

Numbers below indicate the number of participants still under observation (contributing) at each follow-up time point (months) for each outcome and the composite outcome.

© 2026 Beberashvili I et al. *JAMA Network Open*.

eFigure 3. Longitudinal trajectories of normalized dietary protein intake (nDPI) over 15 years of follow-up, stratified by baseline nDPI (<1.0 vs. ≥1.0 g/kg/day). Participants contributed a mean of 1.74 (SD 1.61) annual nDPI assessments (median 1 [IQR 1–2]); ~71% contributed 1 annual assessment and ~13% contributed 2. Mean nDPI values at each year are shown with 95% confidence intervals. Patients with baseline nDPI ≥ 1.0 g/kg/day demonstrated a statistically significant decline over time (slope: −0.019 g/kg/day per year; 95% CI: −0.0304 to −0.0083; $P < 0.001$), whereas patients with lower baseline nDPI showed a non-significant trend (slope: −0.008 g/kg/day per year; 95% CI: −0.0186 to 0.0027; $P = 0.14$).

Numbers below indicate the number of participants still under observation (contributing nDPI measurements) at each follow-up time point (months) by baseline nDPI group; baseline group sizes are shown.

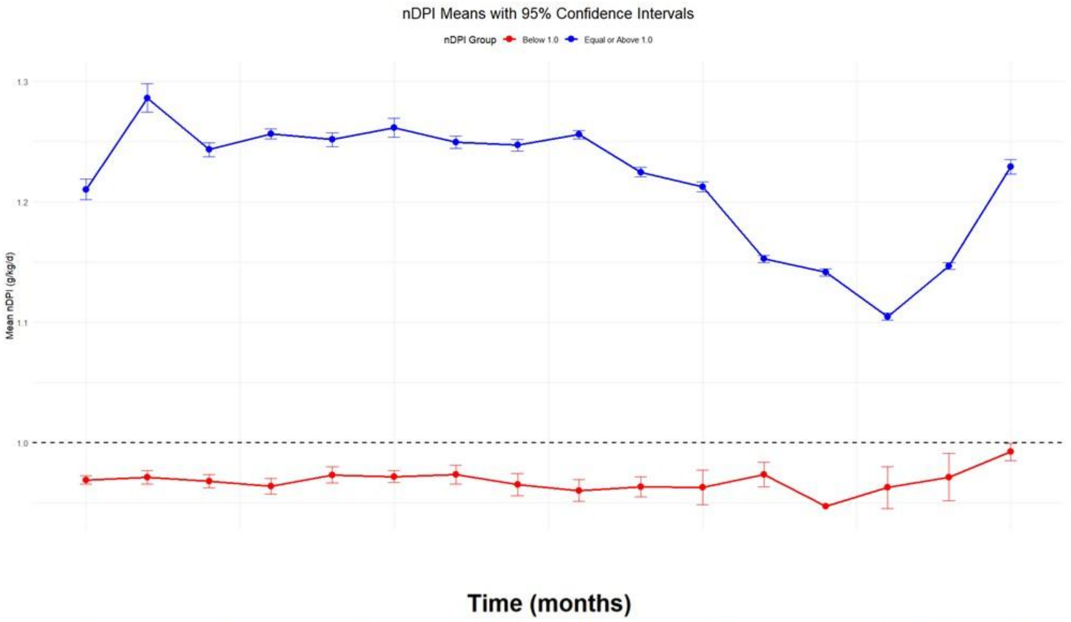

Group	0	12	24	36	48	60	72	84	96	108	120	132	144	156	168	180
nDPI <1.0 (n=321)	321	304	291	270	252	235	219	203	184	158	137	120	93	74	55	42
nDPI ≥1.0 (n=1120)	1120	1065	1015	979	922	860	806	743	657	578	493	431	364	289	215	160

eFigure 3.

© 2026 Beberashvili I et al. *JAMA Network Open*.

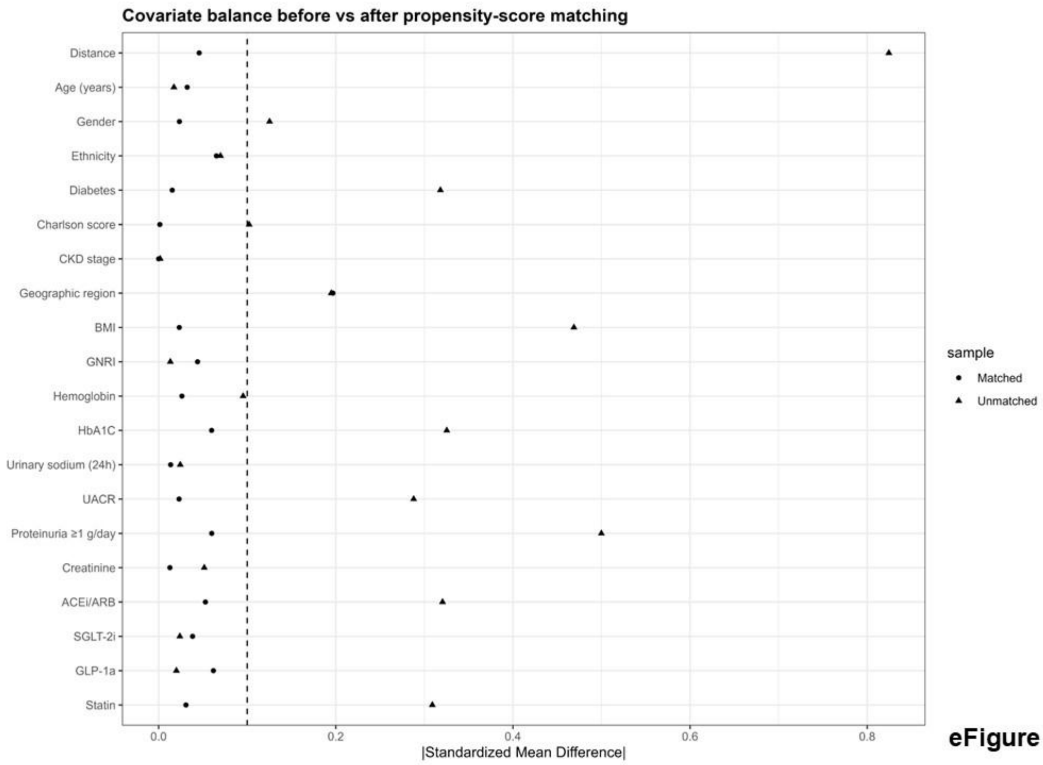

eFigure 4.

eFigure 4. Standardized mean differences (SMDs) for baseline covariates before and after propensity score matching. Each point represents the SMD for a covariate comparing the low and high nDPI groups. Vertical dashed lines at ±0.1 indicate the commonly accepted threshold for acceptable balance. Matching substantially reduced imbalances across covariates, achieving SMDs below 0.1 for all variables.

© 2026 Beberashvili I et al. *JAMA Network Open*.

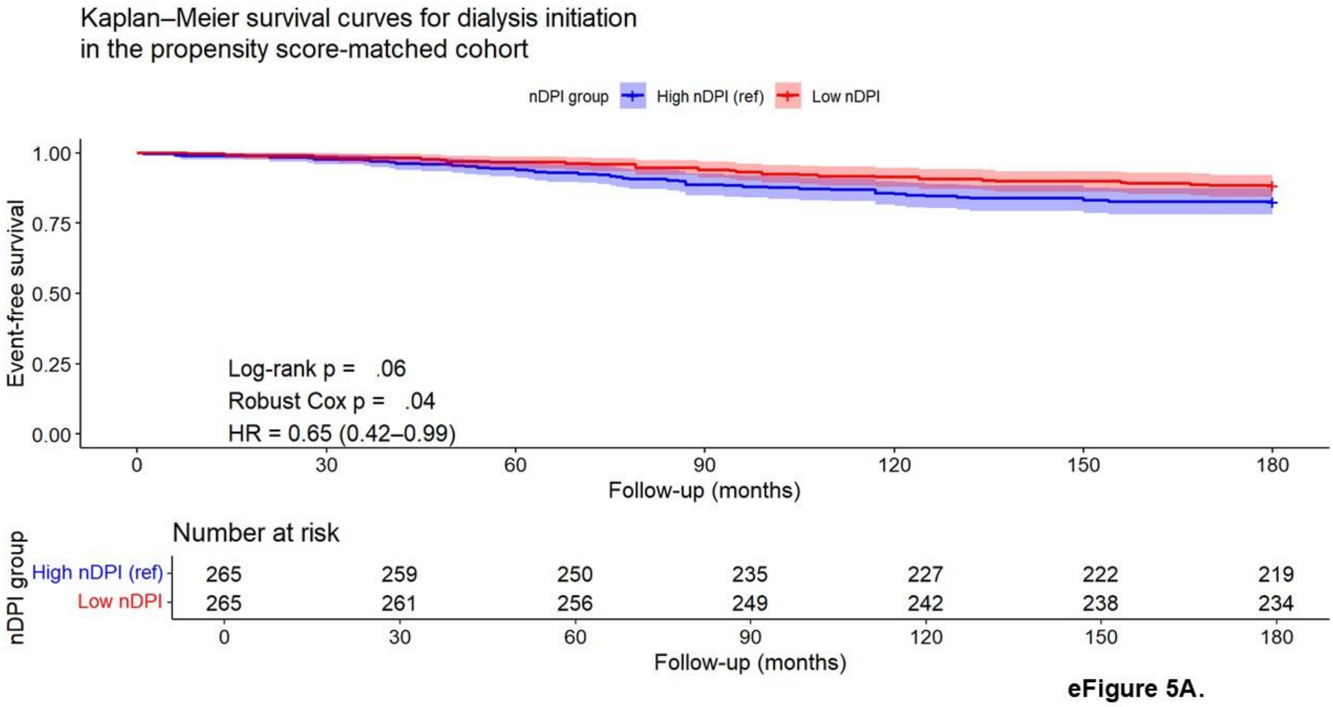

eFigure 5A. Kaplan–Meier survival curves for dialysis initiation by nDPI group. the low nDPI group showed a lower risk (HR 0.65, 95% CI 0.42–0.99; log-rank p =.06; robust Cox p =.04). Numbers at risk are shown. Estimates at late follow-up should be interpreted cautiously due to reduced numbers at risk.

© 2026 Beberashvili I et al. *JAMA Network Open*.

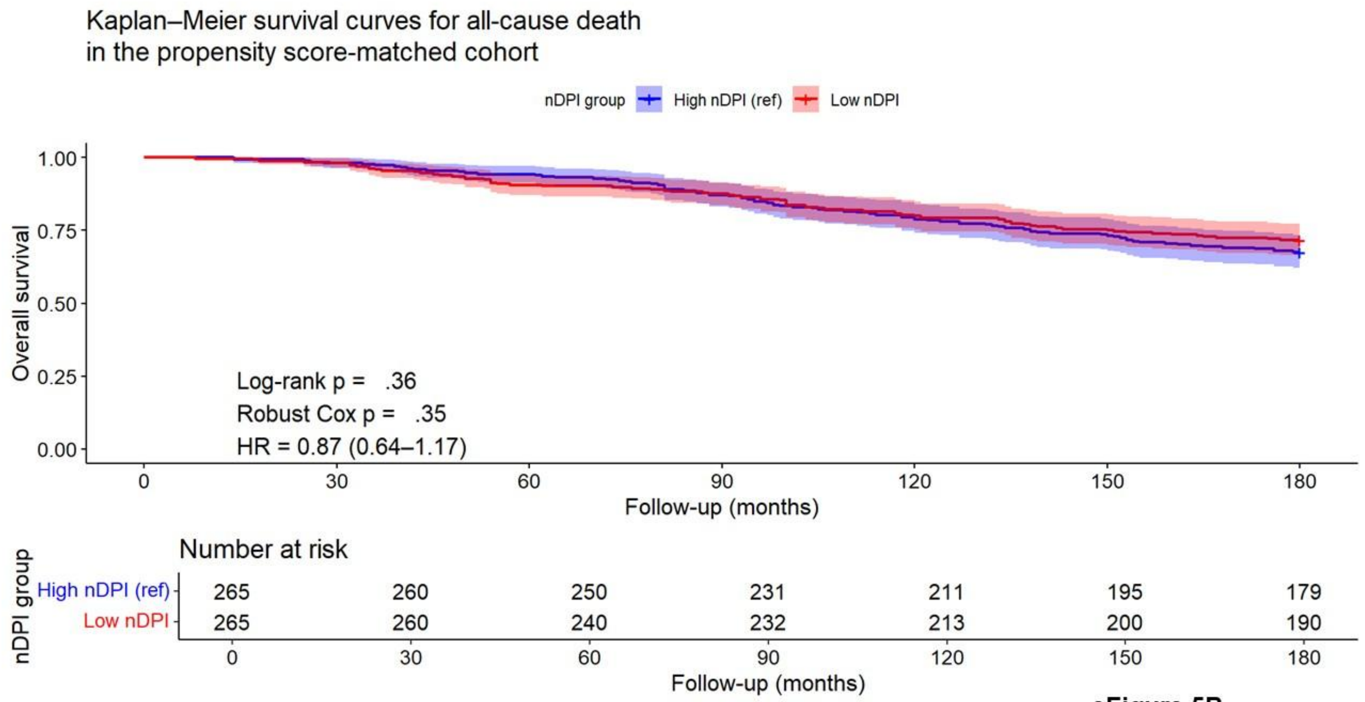

eFigure 5B.

eFigure 5B. Kaplan–Meier survival curves for all-cause mortality by nDPI group. This association did not reach statistical significance (HR 0.87, 95% CI 0.64–1.17; log-rank p =.36; robust Cox p =.35). Numbers at risk are shown. Estimates at late follow-up should be interpreted cautiously due to reduced numbers at risk.

© 2026 Beberashvili I et al. *JAMA Network Open*.

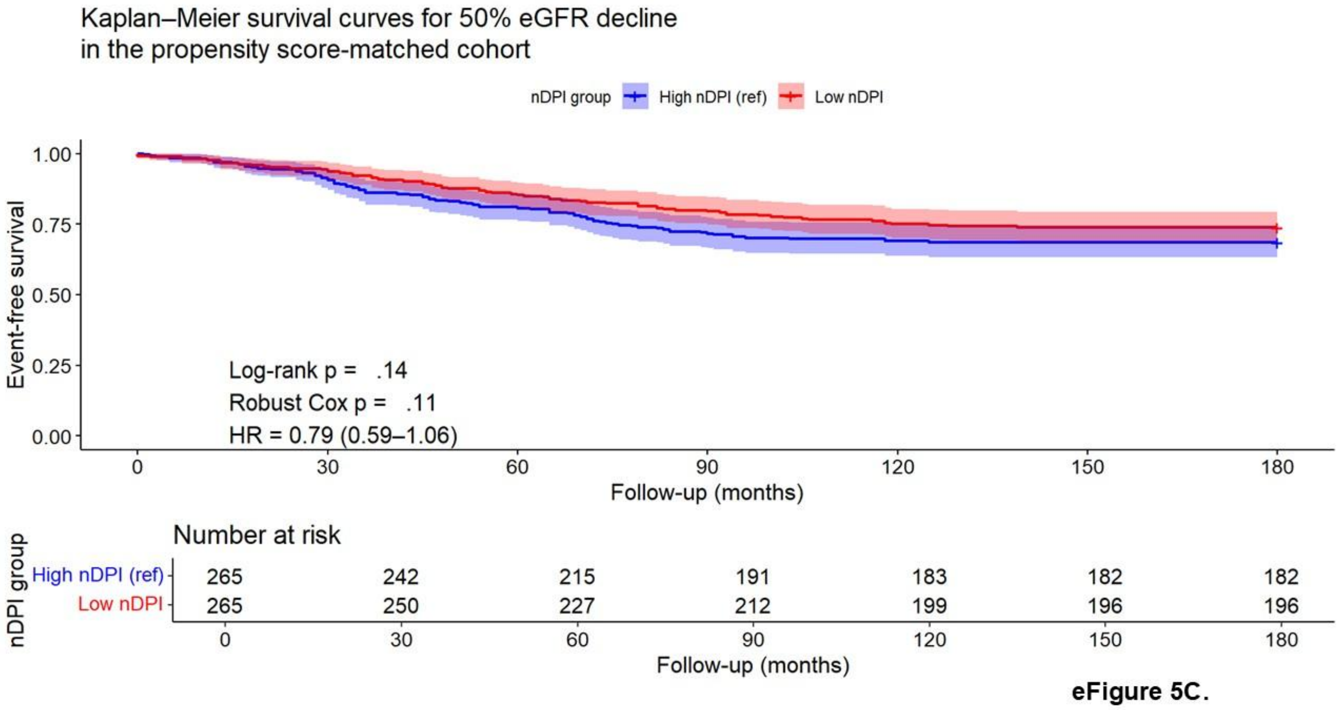

eFigure 5C. Kaplan–Meier survival curves for 50% decline in eGFR by nDPI group. This association did not reach statistical significance (HR 0.79, 95% CI 0.59–1.06; log-rank p =.14; robust Cox p =.11). Numbers at risk are shown. Estimates at late follow-up should be interpreted cautiously due to reduced numbers at risk.

Note, in our study, 'lower nDPI' refers to moderately restricted protein intake (<1.0 g/kg/day), rather than a classical low-protein diet (0.6–0.8 g/kg/day). We therefore use the term 'lower nDPI' throughout to denote this moderate level of restriction.

© 2026 Beberashvili I et al. *JAMA Network Open*.

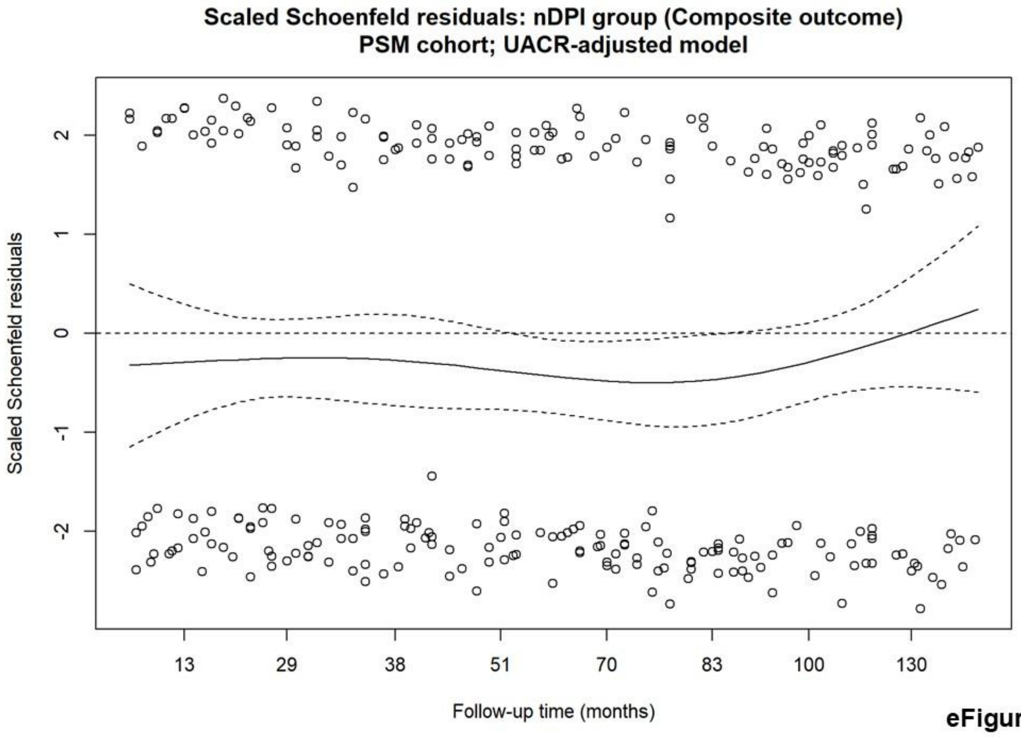

eFigure 6A.

eFigure 6A. Scaled Schoenfeld residuals for the nDPI exposure (composite outcome) in the propensity score–matched cohort. Scaled Schoenfeld residuals are shown for the nDPI group from the UACR-adjusted Cox proportional hazards model. Open circles represent the scaled residuals at observed event times. The solid line depicts the smoothed time-varying coefficient estimate, and dashed lines indicate the approximate 95% confidence limits. The curve remains close to zero over follow-up, supporting no meaningful violation of the proportional hazards assumption for the nDPI exposure in this model.

Brief interpretation: Scaled Schoenfeld residuals for the nDPI group in the UACR-adjusted Cox model (PSM cohort; composite outcome) demonstrate no meaningful departure from proportional hazards. The smoothed residual trend remains near zero throughout follow-up, with only minor fluctuation toward late follow-up where fewer patients remain at risk. Consistent with this visual assessment, the Schoenfeld test did not indicate non-proportionality for the exposure.

© 2026 Beberashvili I et al. *JAMA Network Open*.

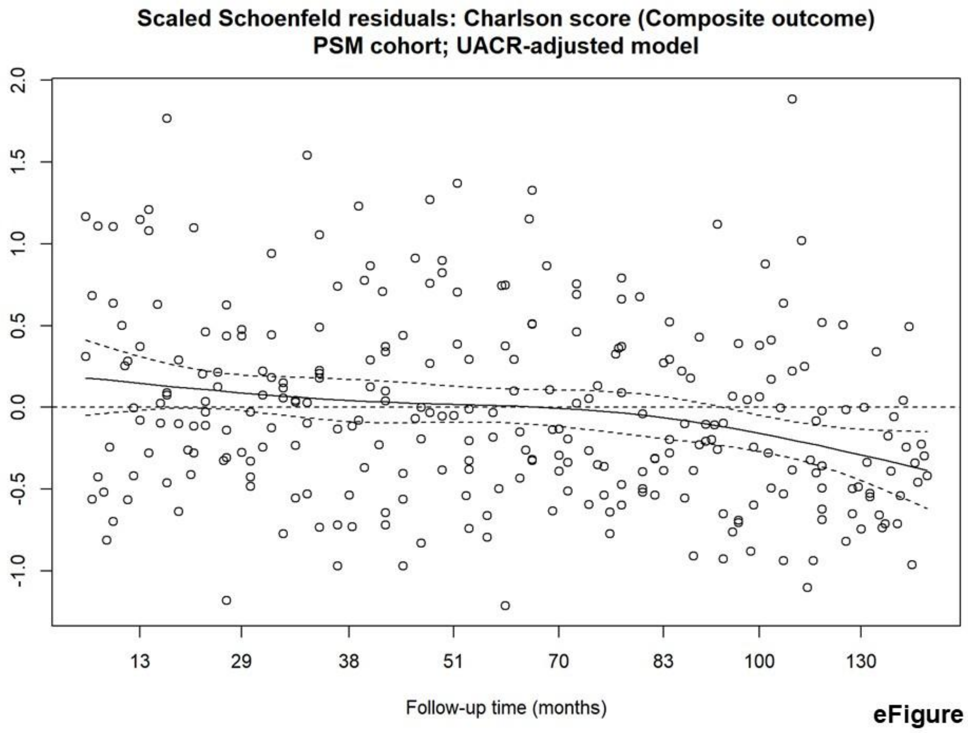

eFigure 6B.

eFigure 6B. Scaled Schoenfeld residuals for Charlson comorbidity score (composite outcome). Shown are scaled Schoenfeld residuals for the Charlson comorbidity score from the UACR-adjusted robust Cox model in the propensity score–matched cohort. Circles represent residuals at event times; the solid line is a smooth estimate of the time-varying coefficient, with dashed lines indicating the approximate 95% confidence band. Systematic deviation of the smooth curve from zero suggests non-proportional hazards for this covariate.

Brief Interpretation: In contrast to the exposure (nDPI group), Charlson comorbidity score showed evidence of non-proportional hazards in the UACR-adjusted model (PH test p≈0.00017), which largely explains the significant global PH test (p≈0.0046). The residual pattern suggests that the effect of comorbidity on the composite outcome changes over follow-up time, whereas the exposure itself did not demonstrate time-dependent effects in this model.

© 2026 Beberashvili I et al. *JAMA Network Open*.

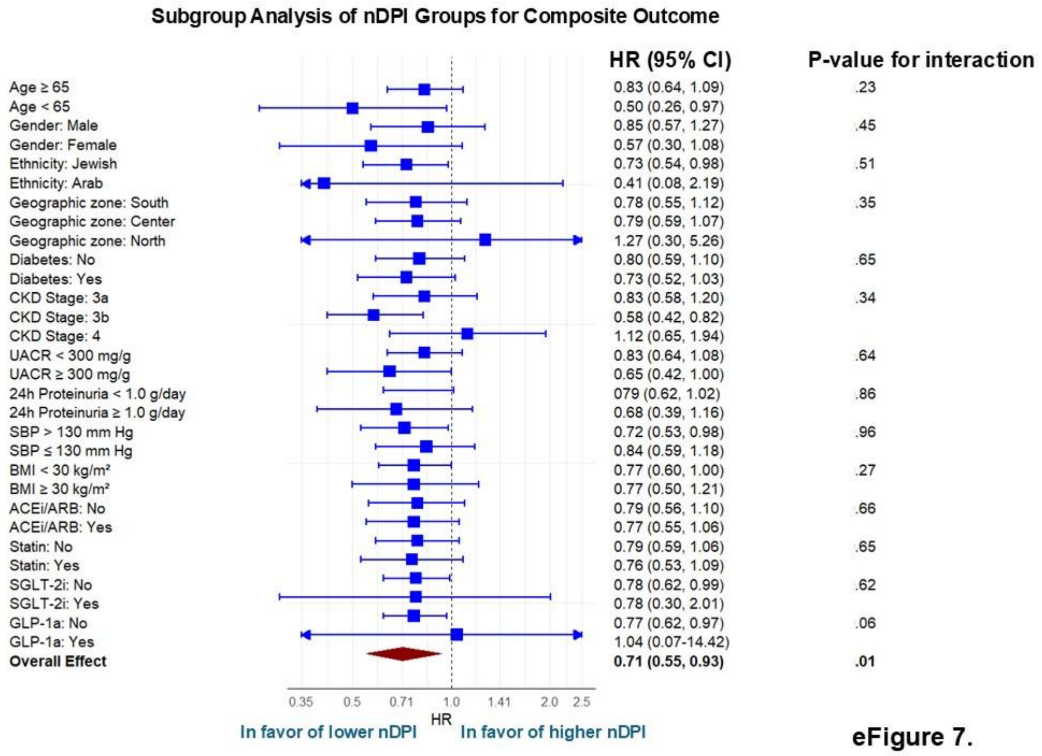

eFigure 7. Subgroup analysis of the association between normalized dietary protein intake (nDPI) and the composite outcome (50% decline in eGFR, dialysis initiation, or all-cause death) in the propensity score-matched cohort. Hazard ratios (HRs) and 95% confidence intervals (CIs) are shown for low nDPI (<1.0 g/kg/day) compared to high nDPI (≥1.0 g/kg/day) across prespecified clinical subgroups. Interaction p-values reflect tests for effect modification. The overall HR was 0.71 (95% CI: 0.55–0.93), indicating a consistent association between low nDPI and lower risk of the composite outcome.

© 2026 Beberashvili I et al. *JAMA Network Open*.

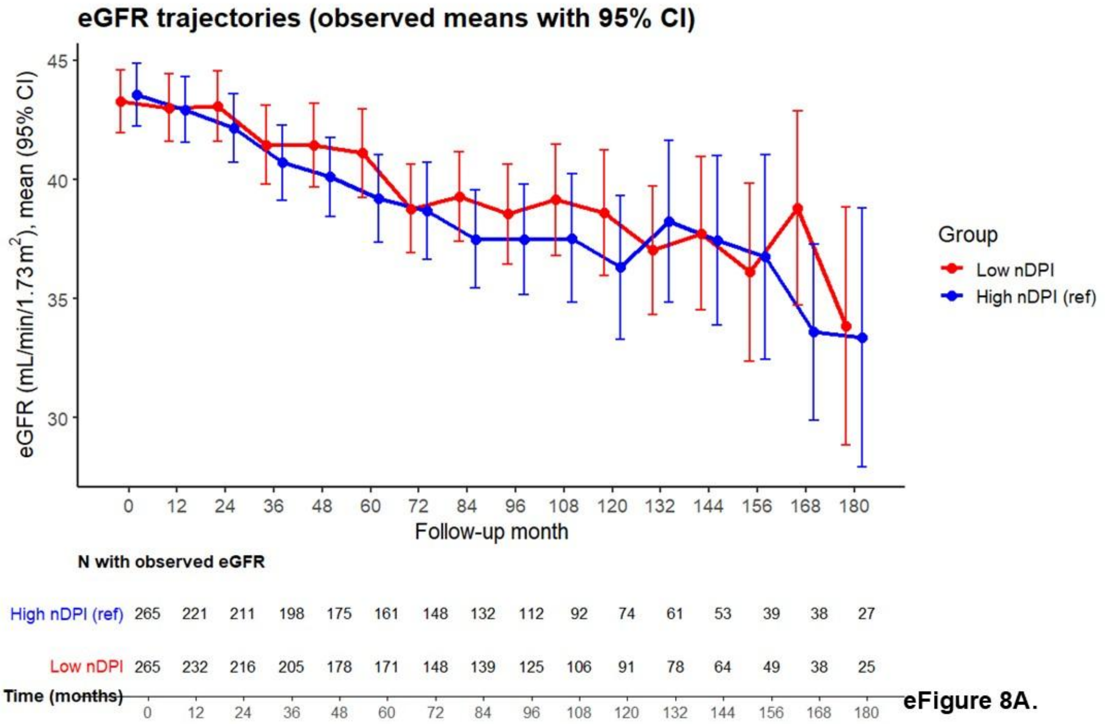

eFigure 8A.

eFigure 8A. Mean eGFR trajectories over 15 years of follow-up in patients with low (<1.0 g/kg/day) and high (≥1.0 g/kg/day) normalized dietary protein intake (nDPI), with 95% confidence intervals. Patients in the low nDPI group exhibited a slower decline in kidney function over time. *Note:* Mean eGFR values represent surviving, uncensored patients at each time point. The average trajectory does not reflect the full distribution, particularly for those who experienced rapid decline or initiated dialysis early.

© 2026 Beberashvili I et al. *JAMA Network Open*.

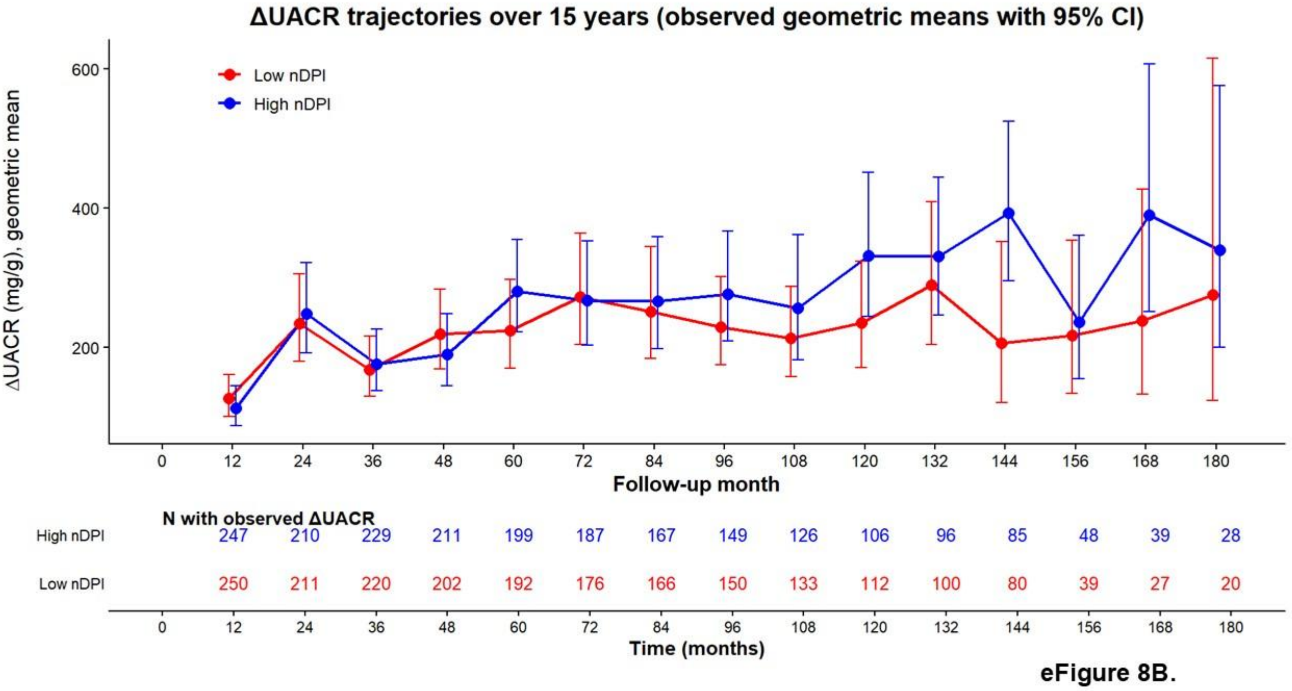

eFigure 8B. Geometric mean changes in urine albumin-to-creatinine ratio (ΔUACR) from baseline over 15 years of follow-up in patients with low (<1.0 g/kg/day) and high (≥1.0 g/kg/day) normalized dietary protein intake (nDPI), with 95% confidence intervals. Patients in the high nDPI group exhibited a steeper increase in albuminuria over time compared to those in the low nDPI group.

Note, in our study, ‘lower nDPI’ refers to moderately restricted protein intake (<1.0 g/kg/day), rather than a classical low-protein diet (0.6–0.8 g/kg/day). We therefore use the term ‘lower nDPI’ throughout to denote this moderate level of restriction.

© 2026 Beberashvili I et al. *JAMA Network Open*.
