# Supplementary material for: Protein Intake and Kidney Outcomes in Nondialysis Chronic Kidney Disease Over 15 Years
Source: JAMA Netw Open. 2026 Apr 28;9(4):e269575. doi: 10.1001/jamanetworkopen.2026.9575 (PMC13126224; doi:10.1001/jamanetworkopen.2026.9575)
Supplement: Supplement 2. — Data Sharing Statement [file jamanetwopen-e269575-s002.pdf]

Data Sharing Statement

Beberashvili. Protein Intake and Kidney Outcomes in Nondialysis Chronic Kidney Disease Over 15 Years. *JAMA Netw Open*. Published April 28, 2026. doi:10.1001/jamanetworkopen.2026.9575

Data
Data available: Yes
Data types: Deidentified participant data
How to access data: Data will be available from the corresponding author upon reasonable request and after IRB approval. Contact: iliabeberashvili63@gmail.com
When available: With publication

Supporting Documents
Document types: None

Additional Information
Who can access the data: To qualified researchers whose proposed use of the data has been approved by the institutional review board.
Types of analyses: For analyses approved by the institutional review board.
Mechanisms of data availability: Researchers must submit a proposal and receive institutional review board approval before access is granted.
Any additional restrictions: No additional restrictions.
